# Research Review: Mechanisms of change and between‐family differences in parenting interventions for children with ADHD – an individual participant data meta‐analysis

**DOI:** 10.1111/jcpp.14120

**Published:** 2025-02-05

**Authors:** Constantina Psyllou, Marjolein Luman, Barbara J. van den Hoofdakker, Saskia Van der Oord, Asma Aghebati, Bianca Boyer, Jan Buitelaar, Andrea Chronis‐Tuscano, David Daley, Tycho J. Dekkers, George J. DuPaul, Gregory A. Fabiano, Maite Ferrin, Nike Franke, Naama Gershy Tsahor, Elizabeth Harvey, Timo Hennig, Sharonne Herbert, Pieter J. Hoekstra, Lee Kern, Jennifer A. Mautone, Amori Yee Mikami, Sébastien Normand, Linda J. Pfiffner, Shizuka Shimabukuro, Satyam Antonio Schramm, Julie B. Schweitzer, Margaret H. Sibley, Edmund Sonuga‐Barke, Catherine Thompson, Margaret J. Thompson, Gail Tripp, Carolyn Webster‐Stratton, Yuhuan Xie, Patty Leijten, Annabeth P. Groenman

**Affiliations:** ^1^ Accare Child Study Center Groningen The Netherlands; ^2^ Department of Child and Adolescent Psychiatry University Medical Center Groningen, University of Groningen Groningen The Netherlands; ^3^ Department of Clinical, Neuro and Developmental Psychology, Clinical Neuropsychology Section Vrije Universiteit Amsterdam Amsterdam The Netherlands; ^4^ Levvel, Academic Center for Child and Adolescent Psychiatry Amsterdam The Netherlands; ^5^ Department of Clinical Psychology and Experimental Psychopathology University of Groningen Groningen The Netherlands; ^6^ Clinical Psychology, Faculty of Psychology and Educational Sciences KU Leuven Leuven Belgium; ^7^ School of Behavioural Sciences and Mental Health, Tehran Institute of Psychiatry Iran University of Medical Sciences Tehran Iran; ^8^ Developmental & Educational Psychology Leiden University Leiden The Netherlands; ^9^ Praktijk Kuin Haarlem The Netherlands; ^10^ Department of Cognitive Neuroscience Radboudumc Nijmegen The Netherlands; ^11^ Karakter Child and Adolescent Psychiatry University Centre Nijmegen The Netherlands; ^12^ Department of Psychology University of Maryland College Park MD USA; ^13^ NTU Psychology, School of Social Science Nottingham Trent University Nottingham UK; ^14^ Department of Child and Adolescent Psychiatry Amsterdam University Medical Centers (AUMC) Amsterdam The Netherlands; ^15^ Department of Psychology University of Amsterdam Amsterdam The Netherlands; ^16^ Department of Education and Human Services Lehigh University Bethlehem PA USA; ^17^ Department of Psychology Florida International University Miami FL USA; ^18^ Department of Child and Adolescent Mental Health Barnet Enfield and Haringey NHS Trust London UK; ^19^ Liggins Institute University of Auckland Auckland New Zealand; ^20^ School of Education The Hebrew University of Jerusalem Jerusalem Israel; ^21^ Department of Psychological & Brain Sciences University of Massachusetts Amherst Amherst MA USA; ^22^ Department of Inclusive Education Faculty of Human Sciences, University of Potsdam Potsdam Germany; ^23^ Department of Advocacy and Public Policy Children's Hospital of Orange County Orange CA USA; ^24^ Department of Child & Adolescent Psychiatry & Behavioural Sciences Children's Hospital of Philadelphia Philadelphia PA USA; ^25^ Department of Psychiatry Perelman School of Medicine at University of Pennsylvania Philadelphia PA USA; ^26^ Department of Psychology University of British Columbia Vancouver BC Canada; ^27^ Département de Psychoéducation et de Psychologie Université du Québec en Outaouais Gatineau QC Canada; ^28^ Institut du Savoir Montfort Hôpital Montfort Ottawa ON Canada; ^29^ Department of Psychiatry and Behavioural Sciences University of California San Francisco San Francisco CA USA; ^30^ Human Developmental Neurobiology Unit Okinawa Institute of Science and Technology (OIST) Graduate University Okinawa Japan; ^31^ Department of Psychiatry and Behavioural Sciences and the MIND Institute University of California, Davis Sacramento CA USA; ^32^ Department of Psychiatry & Behavioural Sciences University of Washington School of Medicine Seattle WA USA; ^33^ Department of Child Psychiatry Institute of Psychiatry, Psychology and Neuroscience, King's College London London UK; ^34^ Department of Psychology University of Hong Kong Pokfulam Hong Kong China; ^35^ Department of Child & Adolescent Psychiatry Aarhus University Aarhus Denmark; ^36^ Institute of Mental Health University of Nottingham Nottingham UK; ^37^ Department of Psychology University of Southampton Southampton UK; ^38^ Specialty Mental Health Program of Asian Health Service Oakland California USA; ^39^ Research Institute of Child Development and Education, University of Amsterdam Amsterdam The Netherlands

**Keywords:** Parent training, ADHD, parenting, meta‐analysis, structural equation modelling

## Abstract

**Background:**

Understanding the mechanisms of change and between‐family differences in behavioural parenting interventions for children with attention‐deficit/hyperactivity disorder (ADHD) may help personalise interventions. Therefore, we examined whether improvements in parenting are associated with changes in child behaviour and functional outcomes, and how these associations vary based on parents' baseline parenting levels.

**Methods:**

We collected individual participant data including 19 randomised controlled trials focusing on children with ADHD (*n* = 1,720). Immediate post‐intervention measures of child ADHD and oppositional behaviour severity, reported by parents and functional impairment reported by either the parent or probably masked clinicians, were treated as outcomes. We estimated pathways from intervention (vs. control) to child outcomes, via immediate post‐intervention parent reports of constructive parenting (e.g. praise), non‐constructive parenting (e.g. physical punishment) and parent–child affection (e.g. warmth), while controlling for baseline values of both child outcomes and parenting levels. Baseline values of each parenting variable were used as moderators of the mediated pathways.

**Results:**

Improvements in parenting behaviours and parent–child affection immediately following the intervention jointly explained concurrent improvements in children's ADHD severity, oppositional behaviour and functional impairment. Furthermore, when reversing the direction of the pathways, improvements in all child outcomes jointly explained improvements in each aspect of parenting. Improvements in non‐constructive parenting and parent–child affection uniquely accounted for intervention effects on functional impairment, especially for families with higher baseline levels of non‐constructive parenting.

**Conclusions:**

Our findings might indicate that improvements in both the behavioural and affective aspects of parenting are associated with concurrent reductions in child behaviour problems and functional impairment. However, more research is necessary to explore the potential causal directionality between parenting and child outcomes. Nonetheless, supporting families with poorer parenting skills may be especially important, as reductions in non‐constructive parenting in these families are linked to stronger treatment effects on child functional impairment.

## Introduction

Behavioural parenting interventions are established as an evidence‐based treatment for children with attention‐deficit/hyperactivity disorder (ADHD). Several meta‐analyses have demonstrated significant intervention effects on parent ratings of ADHD severity and commonly co‐occurring behavioural problems (e.g. Daley et al., 2014; Doffer et al., 2023; Rimestad, Lambek, Zacher Christiansen, & Hougaard, 2019; Sonuga‐Barke et al., 2013). While the effects on masked ADHD outcomes are inconsistent (Daley et al., 2014; Doffer et al., 2023; Sonuga‐Barke et al., 2013), effects on broader masked and unmasked outcomes of impairment in daily functioning, including social relations, family relations and academic achievement have also been demonstrated (Daley et al., 2014; Groenman et al., 2022; Sibley et al., 2023). The core elements of behavioural parenting interventions are grounded in operant learning and social learning theories (Bandura, 1977; Patterson, 1976; Skinner, 1950), and involve breaking coercive patterns of interactions primarily by teaching parents behavioural strategies to encourage more adaptive behaviours and fewer non‐adaptive behaviours in children. Improvements in parenting behaviour and parent–child interactions following such interventions are, therefore, theorised to be a primary mechanism of change in child outcomes. Changes in other aspects of parenting, including parental cognitions and capacity for emotion/behavioural regulation (e.g. accurate attributions, sense of competency and problem‐solving) have also been proposed as potential mechanisms (Katzmann et al., 2017; Rimestad, O'Toole, & Hougaard, 2020; Sanders & Mazzucchelli, 2022). Nevertheless, despite the demonstrated positive effects on both parental and child outcomes (Daley et al., 2014; Dekkers et al., 2022; Doffer et al., 2023; Rimestad et al., 2019; Weber, Kamp‐Becker, Christiansen, & Mingebach, 2019), our understanding of the precise pathways of parenting interventions' effects for children with ADHD remains limited. In the present study, we focused on common pathways related to key parenting aspects targeted by most interventions. This enabled us to conduct a meta‐analysis with individual participant data to delineate their specific role in improving children's behaviour and functional impairment.

Behavioural parenting interventions typically teach parents constructive behavioural parenting strategies designed to develop positive parent–child interactions and disciplinary strategies (Antshel, 2015). For example, they promote the use of contingency management (e.g. positive attention and non‐violent consequences), and/or stimulus control techniques (e.g. providing clear and consistent rules or structuring the child's environment). Some parenting programmes (e.g. Webster‐Stratton, Reid, & Beauchaine, 2011) complement these techniques, by instructing parents to use scaffolding and coaching strategies to teach children social–emotional, organisational and/or academic skills. Also, they encourage parents to avoid non‐constructive parenting strategies (e.g. physical and verbal punishment, permissive or inconsistent discipline, poor monitoring and ineffective communication), and to prevent inadvertently reinforcing oppositional behaviours (Antshel, 2015; Johnston & Jassy, 2007). Hence, improvements in parenting behaviours (i.e. more constructive parenting, less non‐constructive parenting) could be potential pathways associated with improvements in children's ADHD severity, oppositional behaviour and functional impairment.

Along with the core behavioural strategies taught, some programmes specifically aim to build strong parent–child relationships through techniques informed by attachment theories (Scott & Dadds, 2009). These include child‐directed play interactions, where parents learn to identify and positively attend to desired child behaviours, and encourage allocating time for activities with their child (Barkley, 2013). Subsequently, parents may be more attentive and responsive to their child's cues, and able to respond calmly and validate their child's emotions (e.g. ‘It is ok to feel upset’; Rueger, Katz, Risser, & Lovejoy, 2011). This focus on positive affective qualities of parenting may go beyond behavioural strategies and play a crucial role in shaping a healthy, secure attachment between parent and child, which may contribute to positive child outcomes due to reciprocal responsiveness (Scott & Dadds, 2009). For instance, longitudinal studies show that an increase in parental positive regard and expressed emotion predicts a decrease in oppositional behaviour in children at risk of ADHD over the long term (Pauli‐Pott et al., 2021). Therefore, we could assume that increasing positive parent–child affection (i.e. parents responding to children with more positive emotions such as love, warmth and positive involvement versus dysregulated emotions like anger, disappointment and critical or invalidating comments) may also play an important role in explaining intervention effects on child behaviour and functional impairment.

Prior findings from randomised controlled trials (RCTs) provide the strongest evidence that reductions in parent‐rated non‐constructive parenting, as well as composite measures of both constructive and non‐constructive parenting behaviours, serve as mechanisms of change in parent‐rated severity of child behaviour and social functioning (Forehand, Lafko, Parent, & Burt, 2014; Haack, Villodas, McBurnett, Hinshaw, & Pfiffner, 2017; Hanisch, Hautmann, Plück, Eichelberger, & Döpfner, 2014; Mikami, Lerner, Griggs, McGrath, & Calhoun, 2010; Rimestad et al., 2020). To a lesser extent, improvements in outcomes consisting of a combination of constructive parenting behaviours and parent–child affection have been shown to mediate reductions in parent‐rated ADHD severity and oppositional/aggressive behaviours (Hanisch et al., 2014), as well as in parent‐rated social functioning and teacher‐rated academic functioning (Haack et al., 2017). However, parent–child affection, measured by masked observations of parental warmth, has not been found to mediate intervention effects on child behaviour (Hanisch et al., 2014). Importantly, so far, most of the studies analysed different mechanisms of change separately (e.g. Hanisch et al., 2014). Although this approach offers increased statistical power to detect mediation, changes in multiple aspects of parenting often occur simultaneously and interact, and thus should be tested together in one analysis (Patel, Fairchild, & Prinz, 2017). Moreover, previous findings come from RCTs with relatively small sample sizes, which limits our ability to generalise findings across samples and parenting intervention programmes.

To address these limitations, we used data from an Individual Participant Data Meta‐analysis (IPDMA) including multiple international RCTs. This increases the validity and generalisability of results and enables greater statistical power to perform mediation analyses reliably. Additionally, an IPDMA allows a more thorough exploration of interindividual differences in treatment effects. Previous IPDMAs identified participant characteristics including child behaviour severity, single parenthood and maternal depression moderating intervention effects on children's ADHD and oppositional behaviour (e.g. Groenman et al., 2022; Leijten et al., 2020). However, potential parenting factors have not been examined as moderators. Since parents begin the intervention with varying parenting‐related needs that may influence their engagement and capacity for change (Ingoldsby, 2010; Weeland et al., 2023), some families may differ significantly in the pathways mediating their response to treatment. Therefore, we considered baseline levels of parenting behaviour and parent–child affection as moderators of the mediated pathways to child behaviour and impairment. This is a crucial starting point to gain more insight into the processes targeted by parenting interventions and how these may vary across families. Ultimately we may achieve more specific tailoring of programmes to address the unique needs of individual families (Kraemer, Wilson, Fairburn, & Agras, 2002).

In the present study, we applied a moderated mediation analysis (Howe, Beach, Brody, & Wyman, 2016) to (1) test the hypothesis that changes in constructive parenting, non‐constructive parenting and parent–child affection account for intervention effects on children's severity of ADHD, oppositional behaviour and functional impairment and (2) examine if the intervention effects on each child's outcome, via changes in parenting behaviours and parent–child affection vary among families based on parents' baseline levels of these parenting skills.

## Methods

We utilised data from an existing IPDMA dataset, for which the original selection criteria have been previously described (Groenman et al., 2022). The protocol was registered with the international prospective register of systematic reviews (PROSEPRO CRD42022355664), and the study plans were preregistered in AsPredicted (https://aspredicted.org/qz54r.pdf). All deviations from the preregistration are detailed in Appendix S1. We adhered to the PRISMA IPDMA guidelines for reporting (Stewart et al., 2015); a checklist is available in Appendix S2.

### Inclusion criteria

From our IPDMA database, we selected relevant RCTs on behavioural parenting interventions. Behavioural parenting interventions were defined as programmes that primarily focused on parents (with >50% of intervention time allocated to parents) that aimed to change child behaviour predominantly through behavioural techniques targeting parental cognitions, emotions and skills (Evans, Owens, Wymbs, & Ray, 2018). We included trials recruiting children (sample mean age <12 years) with a confirmed ADHD diagnosis or meeting clinical criteria on ADHD questionnaires and/or structured interviews. Trials with a mean sample age below 12 that included some participants older than 12 (e.g. Ferrin et al., 2014, 2020) were not excluded. The studies we included compared the intervention with control conditions, including both passive (e.g. waiting list) and active control arms (e.g. treatment as usual). We excluded studies that compared the intervention to optimised medication treatment, but we included studies that allowed children to be on medication at baseline or during the study period as part of their usual care.

### Study identification and data collection

The included studies had to be published in peer‐reviewed journals in English, German, or Dutch. They were identified through a systematic search conducted up to May 2020, in Medline, CINAHL, PsycINFO, EMBASE+EMBASE CLASSIC, ERIC and Web of Science databases (see Appendix S3 for search terms). The studies were screened independently by two authors using Rayyan (Ouzzani, Hammady, Fedorowicz, & Elmagarmid, 2016), and disagreements were resolved through consensus. Corresponding authors of the eligible trials were contacted via email and personal contacts to request data sharing. The received data were fully anonymized and informed consent was appropriately obtained within each trial. Minor deviations arising from data checks on the provided data were resolved with the corresponding authors.

### Measures

#### Child outcomes

Child outcomes included masked and non‐masked measures. An overview of the instruments included per study is available in the see Table S4A.

*ADHD severity*: Children's ADHD was assessed with parent ratings and observations by a masked rater of inattention and hyperactive–impulsive behaviours.

*Oppositional behaviour*: Oppositional behaviour was assessed via parent ratings of oppositional defiance disorder (ODD) scales and masked observations of disruptive behaviour and non‐compliance.

*Functional impairment*: Functional impairment was evaluated using either parent or probably masked clinician ratings that captured the overall extent of challenge and distress children face in peer relationships, academic functioning and family relationships.

#### Parenting outcomes

Measures of parenting included parent reports on questionnaires that captured parenting practices (see Table S4A for an overview).

*Constructive parenting*: Constructive parenting was broadly conceptualised as non‐violent discipline and behaviours that stimulated or guided the child in developing social–emotional or academic skills. These included scales capturing consequence‐based discipline (e.g. praise, selective attention, logical consequences), antecedent‐based discipline (e.g. setting rules, limits, routines) and effective communication (e.g. reasoning, problem discussion, encouraging expression).

*Non‐constructive parenting*: Non‐constructive parenting was defined as parenting behaviours that do not support the child's development. We used scales of harsh or punitive discipline (e.g. physical and verbal punishment), laxness or lack of follow‐through with discipline and poor monitoring.

*Parent–child affection*: Parent–child affection was defined on a spectrum ranging from intimate parent–child interactions and parental expressed positive emotions to displays of negative or dysregulated emotions. We selected scales measuring parental displays of love, warmth, sensitivity and positive involvement. Measures of parental negative emotions such as anger, disappointment and critical comments were not available in the included studies.

### Data harmonisation

Given that different instruments were used for each measure per study (see Table S4A) and not all studies provided us with item‐level data, we selected the subscales that conceptually fit best with the definitions of each construct, combined with the most frequently used measures across all studies. Specifically for parenting measures, when a subscale of a questionnaire tapped multiple constructs, we assigned this to the parenting aspect that was reflected by the majority of items. The response scales for each construct were harmonised for each individual by converting them into *z* scores, using the baseline standard deviations within studies.

### Risk of bias assessment

The risk of bias in the included studies was assessed independently by two raters (a combination of CP/APG/Master student) with the Cochrane risk of bias tool 2 (Sterne et al., 2019). Each domain of the assessment (i.e. randomisation process, deviations from intended intervention, missing outcome data, measurement of the outcome, selection of the reported results) was evaluated based on the available information reported in the manuscript, using a 3‐point scale (low risk of bias, some concerns, or high risk of bias). In the event of disagreements, consensus was reached through discussion.

### Data analysis

Following our preregistered analytic plans (https://aspredicted.org/qz54r.pdf), we used a one‐stage integrative analysis to merge individual family data from multiple studies. We conducted a moderated mediation analysis using Multilevel Structural Equation Modelling (MSEM) with the ‘lavaan’ package (Rosseel, 2012) in R 4.3.2 (R Core Team, 2022). As the data structure involves nesting of each family (level‐1) within the respective studies (level‐2), we included the study as a cluster‐specific parameter to estimate average intervention effects across families, while adjusting for variability at the study level (Burke, Ensor, & Riley, 2017). We used full information maximum likelihood (FIML) as a parameter estimation. FIML uses available data from all individuals and does not require imputing variables that are completely (systematically) missing across studies, thereby reducing bias in the estimation (Little et al., 2012). Model fit for all estimated models was evaluated using the following a priori guideline (Hu & Bentler, 1999): Comparative fit index (CFI) and Tucker‐Lewis index (TLI) ≥0.95 for excellent fit and ≥0.90 for adequate fit, Root‐mean‐square errors of approximation (RMSEA) ≤0.06 for close fit and ≤0.08 for adequate fit.

#### Modelling approach

**Figure 1:**
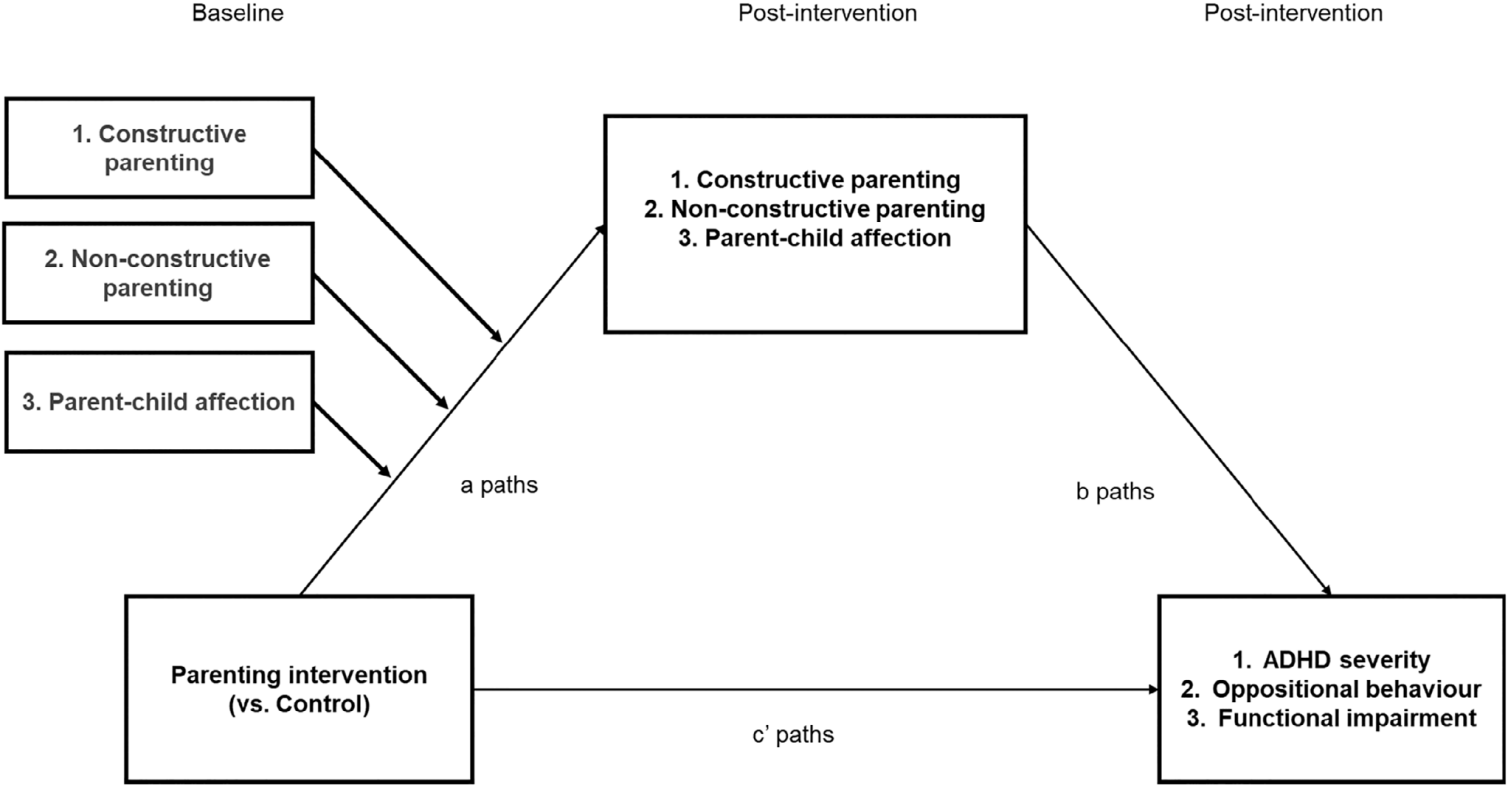
Theoretical model: Baseline moderated mediation. The baseline levels of each parenting domain were used as moderators of each mediated pathway in the model

Mediation analysis in social science typically assumes a time course or cross‐lagged effect between the mediator and outcome variables (Cole & Maxwell, 2003). However, in RCTs of parenting interventions, improvement in child behaviour is expected to occur as soon as parents begin implementing new skills at home, suggesting that changes in parenting and child behaviour may develop in parallel rather than sequentially (Weeland et al., 2018). As a result of this, and since only 42% of trials included follow‐up data with control groups, we used immediate post‐intervention assessments for both parenting and child outcomes, adjusting for their baseline values to capture intervention‐related changes (Valente & MacKinnon, 2017) and accurately examining our moderated mediation model (see Figure 1). We followed Kraemer et al. (2002) guidelines for testing mediation, which required: (1) change in the mediators due to the intervention, (2) temporal precedence of the intervention and (3) a main effect of each mediator on each outcome.

We aimed to use latent constructs of masked and unmasked child outcomes in our model to have a more comprehensive measurement of child behaviour, as assessed by parent ratings and observations by masked raters. However, results from a confirmatory factor analysis (see Table S4B) indicated that masked observations and parent ratings did not represent similar constructs for ADHD severity and oppositional behaviour. Consequently, where possible, we included parent ratings and masked observations as separate outcome variables for ADHD severity and oppositional behaviour in the model.

#### Research question 1: Do intervention effects on parenting predict intervention effects on child outcomes

To answer research question 1, we estimated direct pathways from the intervention arm (parenting intervention: yes/no, with the control condition as the reference group) to post‐intervention measures of child ADHD severity, oppositional behaviour and functional impairment, adjusted for their baseline values. Also, we estimated indirect pathways from intervention to all child outcomes via constructive parenting, non‐constructive parenting and parent–child affection at post‐intervention, adjusted for baseline values. The error terms of parenting and child outcomes were allowed to covary in the model to control for any common causes of change except the intervention. We first assessed the joint indirect effects (i.e. sum of all individual pathways) on each child's outcome. To determine if child outcomes were partially or fully mediated by changes in parenting, we removed the parenting pathways and compared direct intervention effects (*c* paths) to total effects (*c*' paths plus the joint indirect effects). Then, we interpreted the specific indirect effects to test if intervention effects on each parenting aspect were uniquely associated with intervention effects on each child's outcome. Following Hayes (2018), we interpreted all indirect effects, regardless of the significance of the direct intervention effects, using the Sobel test with a significance level of *α* = .05 (Sobel,^1982^).

Given that this model allowed us to establish the association between intervention‐related changes in parenting and child outcomes but not their temporal order or causal role, we reversed the order of pathways in a post hoc analysis (not prespecified) to check if improvements in child outcomes mediated improvements in parenting.

#### Research question 2: Do baseline parenting levels moderate pathways of change across families

To assess research question 2, we allowed the pathways from the intervention arm to each post‐intervention child outcome, via post‐intervention parenting behaviours and parental parent–child affection (i.e. the ‘*a* paths’ × ’*b* paths’ of the parallel mediation model) to be moderated by their baseline values (see Figure 1). We tested the conditional indirect effects (i.e. specific indirect effects of each parenting variable moderated by its baseline levels) with the Johnson‐Neyman Interval test (Hayes, 2018) to probe for a region of significance.

## Results

From a total of 44 studies meeting the inclusion criteria, we received datasets from 21 studies (48%; see Figure S5A for PRISMA flowchart and a comparison between studies that provided data and those that did not). Of these, we excluded two trials focused primarily on interventions targeting adolescents, with a sample mean age above 12 years (*n* = 164, *M*_age_ = 13.83, *SD* = 0.9; Sibley et al., 2014, 2016). These programmes addressed behaviours specific to adolescents (e.g. parent‐teen contracting and organisational skills), which may result in different pathways of change (Forehand et al., 2014). To address this slight deviation from our preregistration, we have presented results including these studies in Table S1. Our final analyses included 1,720 families (1,044 in the intervention group and 676 in the control group) from 19 studies (see Table 1 for a summary of studies that provided data). Children's average age was 7.11 years (*SD* = 2.77 years), ranging from 2.08 to 18.0 years. A total of 20% of children in our sample were on stable medication for ADHD at the start of the intervention. The majority (63%) of the interventions were delivered in a group format, and 21% used a combination of group and individual sessions. A total of 32% of the interventions used a combination of parent, teacher and/or child training (i.e. multimodal interventions). The mean duration of sessions in all parenting programmes was 907 min. A correlation matrix between all outcomes can be found in Table S4C.

**Table 1:** Characteristics of the included studies

	*N*	Intervention, (*n*)	Format	Delivery method	Duration in minutes	Control, (*n*)	Age in years mean (*SD*)	Medication Baseline, *n* yes (%)	Sex male, *n* (%)	SES Low, *n* (%)	SES High, *n* (%)	ODD, *n* yes (%)	Country
Aghebati, Gharraee, Hakim Shoshtari, and Gohari (2014)	27	Triple P (14)	Parent‐only	Group	667	WL (13)	8.04 (1.40)	27 (100)	16 (59)	7 (26)	9 (33)		Iran
Daley and O'Brien (2013)	43	NFPP Self‐Help (24)	Parent‐only	Individual	330	WL (19)	7.28 (1.53)		35 (81)				UK
DuPaul et al. (2018)	45	BPT (15), Online BPT (15)	Parent‐only, Parent‐only	Group, Individual	900, 900	WL (15)	3.84 (0.66)	3 (7)	29 (64)	2 (4)	28 (62)	27 (60)	USA
Fabiano et al. (2012)	55	COACHES (28)	Parent‐only	Group	960	WL (27)	8.51 (1.78)	29 (53)	7 (13)		32 (62)	38 (69)	USA
Ferrin et al. (2020)	69	Psychoeducation (35)	Parent‐only	Group	720	TAU (34)	10.25 (3.12)	37 (65)	60 (90)				UK
Ferrin et al. (2014)	81	Psychoeducation (43)	Parent‐only	Group	1,080	Active (37)	10.6 (3.06)	37 (84)	65 (80)	77 (100)		35 (44)	Spain
Franke, Keown, and Sanders (2020)	53	Triple P Online (27)	Parent‐only	Individual	480	WL (26)	3.97 (0.59)		15 (28)	10 (19)	29 (55)		New Zealand
Herbert, Harvey, Roberts, Wichowski, and Lugo‐Candelas (2013)	31	PYHP (17)	Parent‐only	Group	1,260	WL (14)	4.58 (0.90)		23 (74)	8 (33)	12 (50)	15 (50)	USA
Mautone et al. (2012)	61	FSS (29)	Multimodal	Mixed	980	Active (32)	6.48 (0.6)	15 (25)	44 (72)	3 (5)	48 (79)	18 (29)	USA
Mikami et al. (2010)	62	PFC (32)	Parent‐only	Group	720	WL (30)	8.26 (1.21)	40 (65)	42 (68)	1 (2)	35 (56)	20 (32)	USA
Pfiffner et al. (2007)	69	CLAS (36)	Multimodal	Group	1,260	WL (12), TAU (21)	8.67 (1.16)	2 (3)	46 (67)	1 (1)	49 (73)	16 (23)	USA
Pfiffner et al. (2014)	199	CLAS (74), BPT (74)	Multimodal, Multimodal	Mixed, Mixed	1,260, 1,260	TAU (51)	8.64 (1.16)	7 (4)	116 (58)		160 (81)	31 (16)	USA
Pfiffner et al. (2016)	135	CLS (72)	Multimodal	Mixed	1,125	TAU (63)	8.39 (1.13)	12 (9)	96 (71)	6 (4)	81 (60)	54 (40)	USA
Power et al. (2012)	199	FSS (100)	Multimodal	Mixed	980	Active (99)	9.42 (1.29)	85 (43)	136 (68)	4 (2)	167 (84)	54 (27)	USA
Shimabukuro et al. (2020)	52	Well Parent Japan (28)	Parent‐only	Group	1,560	WL (24)	8.35 (1.68)		42 (82)	15 (30)	14 (28)		Japan
Sonuga‐Barke et al. (2018)	306	IY (131), NFPP (133)	Parent‐only, Parent‐only	Group, Individual	1,620, 1,080	TAU (42)	3.51 (0.58)		224 (73)	133 (44)	41 (14)		UK
Thompson et al. (2009)	41	NFPP (21)	Parent‐only	Individual	600	WL (20)	4.19 (1.06)		31 (76)	28 (85)	2 (6)		UK
Van den Hoofdakker et al. (2007)	94	BPT Groningen (47)	Parent‐only	Group	1,440	TAU (47)	7.43 (1.95)	47 (51)	76 (81)	32 (34)	23 (25)	71 (76)	Netherlands
Webster‐Stratton et al. (2011)	99	IY (49)	Multimodal	Group	2,400	WL (50)	5.36 (0.91)		75 (76)				USA

BPT, Behavioural Parent Training; CLAS, Child Life and Attention Skills; CLS, Collaborative Life Skills; COACHES, Coaching Our Acting‐out Children: Heightening Essential Skills; FSS, Family School Success; IY, Incredible Years; *n*, number of participants in each group; *N*, total number of participants; NFPP, New Forest Parenting Programme; ODD, oppositional defiant disorder; PFC, Parental Friendship Coaching; PYHP, Parenting Your Hyperactive Preschooler; *SD*, standard deviation; SES, socioeconomic status; TAU, treatment as usual; WL, waiting list.

Missing data were primarily due to not all trials measuring every variable of interest. Only three studies (*n* = 385) provided data on masked ADHD severity, and four studies (*n* = 200) on oppositional behaviour, so these were excluded from our analysis (see Appendix S2 for details). Percentages of participants with missing data at baseline, those who did not complete post‐intervention assessments, or who dropped out are presented in Appendix S5 (Figures S5B and S5C). The FIML identified 63 unique patterns of missing values across all variables, summarised visually in Figure S5D.

### Risk of bias assessment

Inter‐rater agreement on the risk of bias was high (*k* = 0.93). Overall, 53% of studies had some concerns in one or more areas, and 47% had a high risk of bias. Most studies (79%) had concerns about the randomisation procedure due to insufficient reporting on how the allocation sequence was generated or whether it was concealed. Regarding outcome measurement, 40% of studies included at least one masked measure of child behaviour or impairment. Although parents were often the main informants, using an active comparison arm reduced the likelihood that parents were aware of any study hypotheses that may have influenced their perceptions and assessments (26% were coded as some concerns, 37% as high risk). Additionally, 33% of trials registered a study protocol but did not preregister their analysis plan, leading to some concerns in the selection of reported results. Detailed risk of bias information is available in the Figures S5E and S5F.

### Modelling results

#### Research question 1: Do intervention effects on parenting predict intervention effects on child outcomes

**Figure 2:**
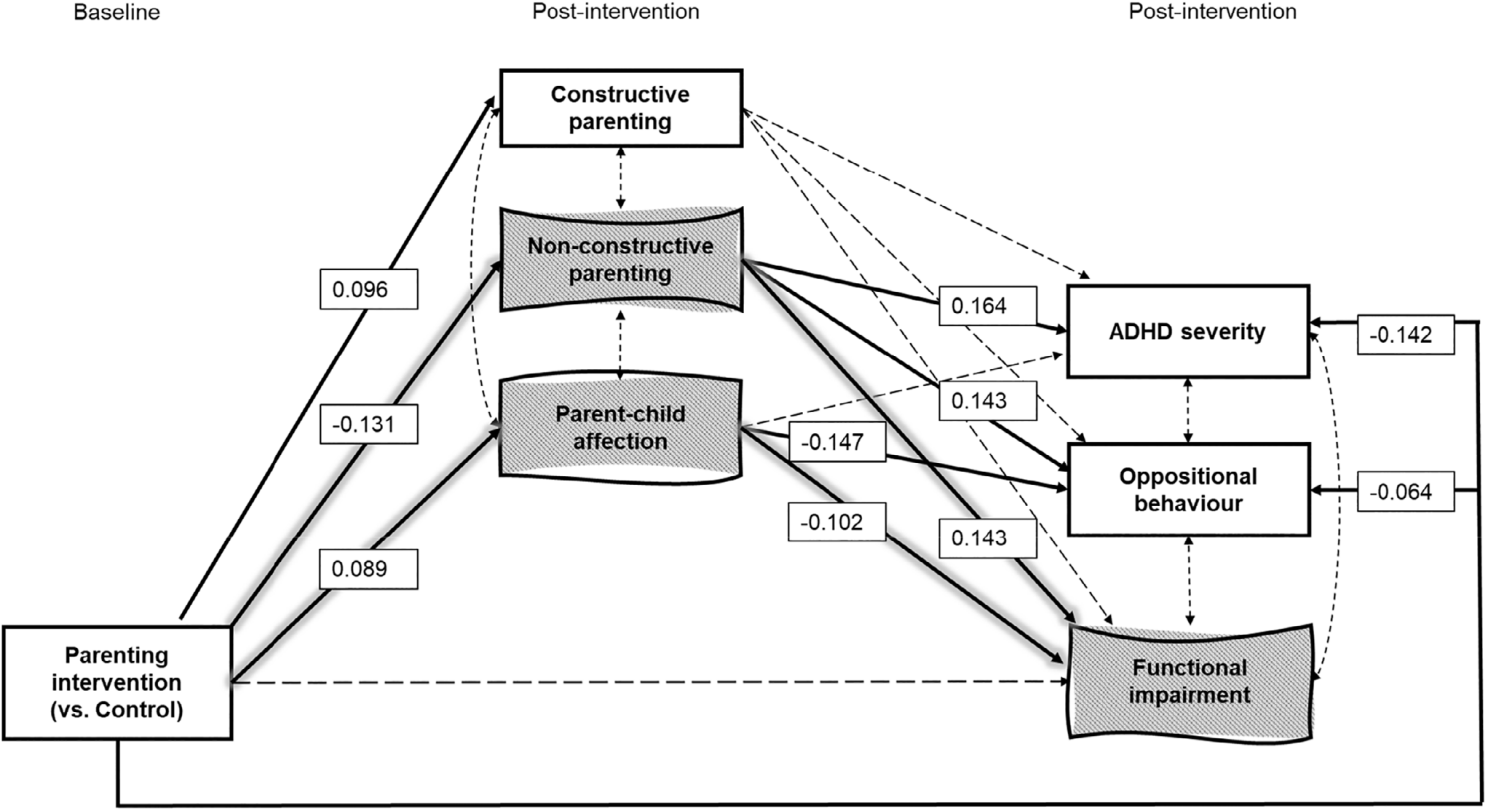
Pathways of change in child outcomes through change in parenting behaviours and parent–child affection. Dashed double‐headed arrows represent covariances between error terms. Dashed arrows depict non‐significant paths, while solid arrows depict significant paths, with their standardised estimates in squares. Corresponding statistics for all paths are provided in Table 2. The grey wavy squares with glowing arrows highlight the specific mediated pathways for changes in functional impairment through changes in non‐constructive parenting and parent–child affection. All post‐intervention measures were controlled for their baseline values to reflect change; these controls are not depicted in the figure for the sake of readability

The parallel mediation model (Figure 2) had good to excellent fit to the data, *χ* ^2^ = 163.755, *df* = 36, *p* < .001, CFI = 0.964, TLI = 0.944, RMSEA = 0.045 (90% CI = 0.038, 0.052). Table 2 describes the estimates for the direct paths (*a*, *b*, *c’* paths) in the mediation model, and the direct intervention effects on each child's outcome (*c* paths) in the model without the parenting pathways.

**Table 2:** Direct paths in the mediation model (*c*’, *b*, *a* Paths) and the model without the parenting pathways (*c* Paths)

Predictors	Direct paths	Child outcomes					
		ADHD severity		Oppositional behaviour		Functional impairment	
		*β* (*SE*)	*p*	*β* (*SE*)	*p*	*β* (*SE*)	*p*
Intervention arm	*c* paths	−0.164 (0.050)	.001	−0.085 (0.026)	.001	−0.060 (0.083)	.467
Intervention arm	*c*′ paths	−0.142 (0.055)	.010	−0.064 (0.028)	.025	−0.042 (0.079)	.594
Constructive parenting	*b*_1_ paths	−0.031 (0.034)	.355	0.019 (0.028)	.495	−0.072 (0.054)	.179
Non–constructive parenting	*b*_2_ paths	0.164 (0.060)	.006	0.143 (0.044)	.001	0.143 (0.033)	<.001
Parent–child affection	*b*_3_ paths	−0.081 (0.045)	.075	−0.147 (0.042)	.001	−0.102 (0.036)	.005

		Parenting outcomes					
		Constructive parenting		Non–constructive parenting		Parent–child affection	
Intervention arm	*a* paths	0.096 (0.034)	.005	−0.131 (0.060)	.028	0.089 (0.035)	.010

Intervention arm was modelled using a dummy code (parenting intervention: yes/no, with the control condition as the reference group). The significance of effects was determined with a significance level of *α* = .05. *β*, standardised path estimate; SE, standard error.

For intervention effects in parent‐reported ADHD severity, significant partial mediation was observed through joint improvements in constructive parenting, non‐constructive parenting and parent–child affection (*β* ab_total1_ = −0.031, *p* = .039). These joint parenting pathways accounted for 18% of the total intervention effect (*β*_total1_ = −0.172, *p* < .001). For intervention effects in parent‐reported oppositional behaviour, significant partial mediation was observed through joint improvements in all aspects of parenting (*β* ab_total2_ = −0.030, *p* = .018), with these pathways explaining 32% of the total intervention effect (*β*_total2_ = −0.094, *p* < .001). For intervention effects in functional impairment as reported by parents or clinicians, significant mediation was observed through joint improvements in all aspects of parenting (*β* ab_total3_ = −0.035, *p* = .002), which explained 32% of the total intervention effect (*β*_total3_ = −0.078, *p* = .334). We did not have sufficient evidence to determine whether this mediation effect was partial or full because the direct intervention effect on functional impairment in the model without the parenting pathways was not significant (*β* ^c3^ = −0.060, *p* = .467; see Table 2). This lack of significance may be due to suppression effects from including the covariance between the error terms of all child outcomes in the model. Post hoc analysis of the reverse mediation model indicated that intervention effects on constructive parenting (*β* ab_total1_ = 0.01, *p* = .045), non‐constructive parenting (*β* ab_total2_ = −0.032, *p* = .002) and parent–child affection (*β* ab_total3_ = 0.031, *p* = .031) were also explained by joint improvements in child ADHD severity, oppositional behaviour and functional impairment (see Appendix S6).

When considering unique changes in constructive parenting, non‐constructive parenting and parent–child affection, there was no evidence of specific mediation for improvements in child ADHD severity and oppositional behaviour (see Table 3). However, there was evidence of specific mediation for a reduction in functional impairment through unique improvements in non‐constructive parenting (*β* ab_13_ = −0.019, *p* = .041) and parent–child affection (*β* ab_33_ = −0.009, *p* = .020). The reverse mediation analysis did not provide evidence of specific mediation for improvements in non‐constructive parenting and parent–child affection via a reduction in functional impairment (see Figure S5G and Appendix S6 for all results).

**Table 3:** Specific and joint indirect effects in the mediation model and conditional indirect effects in the moderated mediation model

		Child outcomes					
		ADHD severity		Oppositional behaviour		Functional impairment	
Parenting pathways	Indirect paths	*β* (*SE*)	*p*	*β* (*SE*)	*p*	*β* (*SE*)	*p*
Constructive parenting	Specific indirect	−0.003 (0.004)	.421	0.002 (0.003)	.505	−0.007 (0.005)	.163
	Conditional indirect	0.005 (0.005)	.264	−0.003 (0.004)	.498	0.010 (0.010)	.321
Non–constructive parenting	Specific indirect	0.022 (0.014)	.111	−0.019 (0.012)	.113	−0.019 (0.009)	.041
	Conditional indirect	−0.016 (0.010)	.118	−0.015 (0.009)	.098	−0.015 (0.007)	.044
Parent–child affection	Specific indirect	−0.007 (0.006)	.219	−0.013 (0.008)	.088	−0.009 (0.004)	.020
	Conditional indirect	0.003 (0.004)	.340	0.008 (0.007)	.282	0.006 (0.005)	.299
Joint indirect		−0.032 (0.015)	.035	−0.030 (0.013)	.018	−0.035 (0.011)	.002
Total		−0.174 (0.049)	<.001	−0.094 (0.026)	<.001	−0.078 (0.081)	.334

Specific indirect effects represent the direct pathway from the intervention arm on each parenting variable (*a* paths) multiplied by the pathway of each parenting variable on each child's outcome (*b* paths). The joint indirect effects represent the joint mediated pathways via all parenting variables in the mediation model. Total effects represent the direct *c* path from intervention to each child outcome in addition to the joint indirect effect. The conditional indirect effects of the moderated mediation model represent the parenting pathways that are moderated by the baseline levels of each parenting variable. *β =* standardised path estimate; SE = standard error. Intervention arm was modelled using a dummy code (parenting intervention: yes/no, with the control condition as the reference group). The significance of effects was determined with a significance level of *α* = .05.

#### Research question 2: Do baseline parenting levels moderate pathways of change across families

The baseline moderated mediation model had an excellent fit to the data, *χ* ^2^ = 172.366, *df* = 51, *p* < .001, CFI = 0.965, TLI = 0.949, RMSEA = 0.037 (90% CI = 0.031–0.043). Baseline levels of non‐constructive parenting significantly moderated the effect of the intervention on functional impairment via intervention‐related changes in non‐constructive parenting (*β* conditional indirect effect = −0.015, *p* = .044). As revealed by the Johnson‐Neyman Interval test, a reduction in non‐constructive parenting at post‐intervention was more strongly linked to improvement in functional impairment immediately following the interventions for parents whose non‐constructive parenting was above‐average levels at baseline (z score >0). None of the other mediated pathways on each of the child outcomes varied by parents' baseline levels of parenting (see Table 3).

## Discussion

In this study, we examined individual differences in the pathways leading to parenting intervention effects for children with ADHD. We showed that joint improvements in parent reports of constructive and non‐constructive parenting behaviours and parent–child affection were associated with simultaneous improvements in all child outcomes, including ADHD severity and oppositional behaviour as reported by parents, and functional impairment as reported by parents or probably masked clinicians. Although the temporal order of effects was not directly evaluated in our analysis, these findings could indicate that improvements in parenting behaviours and parent–child affection interact or work together to explain improvements in child outcomes. The joint pathways accounted for a slightly smaller proportion of the total intervention effect on ADHD severity (18%) compared to oppositional behaviour and functional impairment (32% each). This finding could suggest that reducing children's ADHD severity through changes in parenting alone may be more challenging. Furthermore, it is also possible that improvements in certain child behaviours may be necessary, as reversing the order of pathways in the model revealed that joint improvements in all child outcomes also contributed to changes in each aspect of parenting.

Similar to previous findings (e.g. Booster, Mautone, Nissley‐Tsiopinis, Van Dyke, & Power, 2016; Dose, Hautmann, Bürger, Schürmann, & Döpfner, 2021; Haack et al., 2017), we found that a reduction in non‐constructive parenting, but not an increase in constructive parenting, uniquely accounted for intervention effects on functional impairment. One possibility is that reducing non‐constructive parenting may be particularly beneficial because it may result in fewer instances of coercive family interactions that directly impair family functioning (Deault, 2010). Also, children may source less non‐constructive information from their interactions with their parents and have more opportunities to learn skills at home that can further reduce impairments in areas such as academic and social functioning (Tarver, Daley, & Sayal, 2015). However, as all parenting programmes target constructive parenting behaviours, improvements in these could be more relevant for explaining intervention effects on distinct positive child outcomes, not examined in the current study, such as planning and organisation skills, or social and emotional skills (Webster‐Stratton et al., 2011).

Improved parent–child affection also uniquely accounted for intervention effects on functional impairment. This may be explained in different ways. Firstly, parent–child affection may be especially important for children's functioning at home, in schools and with peers (Tarver et al., 2015). This is consistent with prior results showing an indirect intervention effect via improvements in parental warmth and involvement on children's social and organisational skills impairment (Haack et al., 2017). Second, by improving parent–child affection parents may develop more compassion for their child's behaviour, which may translate towards less reported functional impairment (Shelleby & Ogg, 2020; Shenaar‐Golan, Wald, & Yatzkar, 2021). Nevertheless, parent–child affection operates in combination with certain parenting behaviours (Crandall, Deater‐Deckard, & Riley, 2015; Rueger et al., 2011). For example, parents may offer praise with more warmth and enthusiasm or praise may be more meaningful and effective for children who feel closer to their parents following a parenting intervention (Owen, Slep, & Heyman, 2012). This may be particularly the case for parenting programmes that include elements aimed at improving the parent–child relationship (Phillips et al., 2024). Therefore, more research is needed on the working mechanism of specific parenting intervention components (Kaehler, Jacobs, & Jones, 2016).

Our results showed unique associations for intervention effects on non‐constructive parenting and parent–child affection with intervention effects on functional impairment, but not on ADHD severity and oppositional behaviour. This divergence in findings may be attributable to the fact that the daily impairments associated with ADHD are often the main concern of parents and thus the main target of treatment (Chronis‐Tuscano, Chacko, Fabiano, Wymbs, & Pelham, 2004). The results suggest that improvements in non‐constructive parenting and parent–child affection alone are sufficient to account for the treatment effect on functional impairment. In contrast, a combination of changes across several aspects of parenting may better explain improvements in ADHD severity and oppositional behaviour.

Although joint improvements in all parenting practices significantly accounted for intervention effects on all child outcomes, there were still significant intervention effects on children's severity of ADHD and oppositional behaviour that remained unexplained. This suggests there might be changes in parenting practices or third factors that we did not account for in our model, which may also be associated with improvement in child behaviour. For example, teaching parents to apply emotion coaching strategies could be particularly important in mitigating ADHD and oppositional behaviours, as children with ADHD often experience emotional dysregulation and heightened sensitivity to rewards and punishments (Breaux, McQuade, Harvey, & Zakarian, 2018; Van der Oord & Tripp, 2020). Child behaviour severity could be improved by training children in social skills, problem‐solving and organisational strategies (Sibley et al., 2023). This is particularly relevant for the multimodal interventions, where we found significant parenting pathways associated with improved functional impairment but not behaviour severity (see sensitivity analysis in Appendix S7). Additionally, factors related to the method and quality of treatment delivery (e.g. group format, practitioner flexibility and empathy) might produce non‐specific effects that are associated with child behaviour improvements (Leitão, Francisco, Gaspar, & Seabra‐Santos, 2023; Sanders & Mazzucchelli, 2022). For example, by empowering parents, reducing isolation, increasing sense of competence and decreasing depression and stress (Leitão, Pereira, Santos, Gaspar, & Seabra‐Santos, 2022; Levac, McCay, Merka, & Reddon‐D'Arcy, 2008; Mathijs et al., 2024).

The fact that we used post‐intervention assessments in our analyses prevents us from establishing temporal ordering of change. Interestingly, when we reversed the order of the pathways in our model, we did not detect a specific pathway in which improvements in functional impairment predicted improvements in parenting behaviours and parent–child affection. However, improvements in all child outcomes jointly predicted intervention effects on non‐constructive parenting and parent–child affection. Thus, we cannot rule out the possibility that improvements in children's behaviour may precede improvements in some parenting outcomes. Future RCTs studies should adopt time‐based approaches such as cross‐lagged modelling (Wu, Carroll, & Chen, 2018) with mid‐treatment data points to provide a clearer perspective on the causal directionality between parenting and child outcomes. Also, follow‐up studies with repeated measurements in a longer timespan are necessary to determine if changes in certain parenting practices and/or child behaviours might be necessary to enable a cascade of changes in other outcomes (e.g. Novick et al., 2023; Smit, Mikami, & Normand, 2022).

In our attempt to detect subgroups of families who may improve through different mechanisms, we found that intervention effects on non‐constructive parenting were more strongly associated with intervention effects on functional impairment for families who initially displayed above‐average levels of non‐constructive parenting (*z* scores > 0). This aligns with evidence suggesting that parenting interventions are more beneficial for improving child behaviour in families with particularly high levels of non‐constructive parenting at baseline (Beauchaine, Webster‐Stratton, & Reid, 2005). Our results suggest that families facing more challenges at baseline may benefit most from changes in non‐constructive parenting, possibly due to greater need and potential for improvement. However, this could also reflect regression to the mean, where the scores of individuals with the highest levels of baseline non‐constructive parenting are more likely to become less extreme following intervention. Additionally, factors beyond the scope of our study, such as parental well‐being (e.g. stress, ADHD, depression) and child behaviour severity or medication status could have confounded the moderation findings, because they may interact with parenting (Chronis‐Tuscano et al., 2011; Fenesy, Teh, & Lee, 2019; Kaiser, McBurnett, & Pfiffner, 2011; Mikami et al., 2020), and may also affect attendance and skills implementation (Melendez‐Torres et al., 2024; Reyno & McGrath, 2006). More efforts are needed in future studies to identify subgroups of families, based on specific combinations of baseline characteristics, who benefit most through different mechanisms.

### Strengths and limitations

To our knowledge, the presented work is the first to assess individual differences in the pathways associated with parenting interventions' outcomes for children with ADHD in a large (*n* = 1,721) international sample using IPDMA. Despite several merits, our findings should be seen in light of some limitations.

First, we could not delineate the time sequence of the pathways of change. Especially for programmes that include both child‐directed and parent‐directed components, improvements in child behaviour might precede or occur independently of improvements in parenting, potentially creating a cycle where both improve simultaneously. Second, while using broad parenting constructs may have facilitated the detection of mediation (e.g. Forehand et al., 2014), it limited our ability to identify specific techniques (e.g. praise, planned ignoring, time‐out, logical consequences) tailored to a child's age and developmental stage, which could be associated with treatment effects in different subgroups (e.g. preschoolers, school‐age children and adolescents). Third, we did not have sufficient data for masked outcomes, thus we used parental ratings of their own and their child's behaviour. This may have inflated associations due to shared informant bias or effort justification. However, parental perceptions of their child's behaviour are clinically relevant as they often drive the decision to seek support (O'Connor et al., 2015). Fourth, a common challenge in IPDMA is the reliance on measures available in each individual study. Not all trials included the same measures, which prevented the calculation of treatment effects for every outcome across all trials (e.g. 32% did not include data on parenting and 63% did not include data on functional impairment). To address missing data, we had to rely on FIML, a standard approach in RCTs (Little et al., 2012). Additionally, information on treatment fidelity (e.g. dosage, practitioner skills, adherence to treatment protocols) and parental adherence (e.g. attendance, skill implementation) were unavailable. As these factors may be important for treatment success (Eames et al., 2009; Leitão et al., 2022), they should be considered in future research investigating pathways of change in parenting interventions. Fifth, results should be interpreted keeping in mind the potential biases present in the studies included as for 47% of studies the risk of bias was high in at least one domain. Nevertheless, given the challenges of blinding group allocation and common issues with handling dropouts and attrition in parenting intervention trials, achieving a low risk of bias is difficult. Finally, since 74% of the data originate from English‐speaking countries, the generalisability of our findings to other cultural contexts may be limited. However, there was some ethnic diversity within the trials. Future research should aim for greater inclusivity to better understand how to tailor interventions to meet the diverse needs of families worldwide.

## Conclusion

Our findings suggest that changes in both behavioural and affective components of parenting may be necessary to explain treatment effects on child behaviour and functional impairment. Although we cannot rule out the possibility that improvements in child outcomes precede improvements in parenting, our findings provide insight into the complex mechanisms of parenting interventions and how these may vary across families based on their initial parenting skills. For families with higher initial levels of non‐constructive parenting, there was a stronger association between improvements in non‐constructive parenting and functional impairment compared to families with lower levels of non‐constructive parenting. This finding could guide clinicians in better supporting families who struggle to provide a constructive context for their children's development.

## Author contributions

*Conceptualisation*: Groenman, Hoekstra, Psyllou, Buitelaar, Luman, Leijten, Van der Oord and Van den Hoofdakker. *Data curation—Collection and management of data*: Psyllou, Luman, Van der Oord, Leijten, Dekkers and Groenman. *Data curation—Contribution of data*: Aghebati, Boyer, Chronis‐Tuscano, Daley, DuPaul, Fabiano, Ferrin, Franke, Gershy Tsahor, Harvey, Hennig, Herbert, Lee Kern, Mautone, Mikami, Normand, Pfiffner, Shimabukuro, Schramm, Schweitzer, Sibley, Sonuga‐Barke, C. Thompson, M. Thompson, Tripp, Webster‐Stratton and Xie. *Formal analysis*: Psyllou and Groenman. *Funding acquisition*: Groenman, Van den Hoofdakker, Hoekstra, Van der Oord, Leijten, Luman, Dekkers. Authors that contributed data were responsible for funding the acquisition of the individual trials. *Investigation*: Psyllou, Luman, Van den Hoofdakker, Van der Oord, Leijten, Groenman. Authors that contributed data were responsible for the investigation of the individual trials. *Methodology*: Psyllou, Luman, Van den Hoofdakker, Van der Oord, Leijten, Groenman. Authors that contributed data were responsible for the methodology of the individual trials. *Project administration*: Groenman. *Resources*: Hoekstra. *Supervision*: Luman, Van den Hoofdakker, Van der Oord, Leijten and Groenman. *Visualisation*: Psyllou. *Writing—Original draft*: Psyllou, Luman, Van den Hoofdakker, Van der Oord, Leijten and Groenman. *Writing—Review and editing*: Aghebati, Boyer, Buitelaar, Chronis‐Tuscano, Daley, Dekkers, DuPaul, Fabiano, Ferrin, Franke, Gershy Tsahor, Harvey, Hennig, Herbert, Lee Kern, Mautone, Mikami, Normand, Pfiffner, Shimabukuro, Schramm, Schweitzer, Sibley, Sonuga‐Barke, C. Thompson, M. Thompson, Tripp, Webster‐Stratton and Xie.

## Ethical considerations

The received data were fully anonymized and informed consent from participants was appropriately obtained within each trial. Investigators of each individual trial were responsible for receiving ethics approval for conducting research with human participants.

**Key points**

- Behavioural parenting interventions are effective for children with ADHD, but their mechanisms of change are not well understood and may vary among families.
- In this IPDMA, improvements in constructive parenting, non‐constructive parenting and parent–child affection jointly account for intervention effects on ADHD severity, oppositional behaviour and functional impairment in children.
- It seems important to specifically support families with poorer parenting skills because for these families, improvements in functional impairment of their child relate to improvements in parenting skills.
- Future studies should incorporate more frequent measurements and adopt time‐based approaches such as cross‐lagged modelling to explore further the plausibility of causal directionality among parenting and child outcomes.

## Supporting information


**Appendix S1.** Changes from preregistration.
**Table S1.** Baseline targeted moderated mediation analysis including the additional adolescent studies.
**Appendix S2.** PRISMA checklist.
**Appendix S3.** Search terms per database.
**Appendix S4.** Supplementary tables.
**Table S4A.** Outcome measures used per included study.
**Table S4B.** Confirmatory factor model for masked and unmasked child behaviour.
**Table S4C.** Correlation matrix between outcomes.
**Appendix S5.** Supplementary figures.
**Figure S5A.** PRISMA 2009 flow diagram.
**Figure S5B.** Percentages of missing data on parenting outcomes within studies.
**Figure S5C.** Percentages of missing data on child outcomes within studies.
**Figure S5D.** Missing data patterns on the post‐intervention measures across studies.
**Figure S5E.** Risk of bias studies.
**Figure S5F.** Risk of bias per study.
**Figure S5G.** Reverse mediation model.
**Appendix S6.** Post hoc analysis results for the reverse mediation.
**Appendix S7.** Sensitivity mediation analysis results on the multimodal interventions.

## Data Availability

The data that support the findings of this study are available from the corresponding authors upon reasonable request.
