# Supplementary material for: Research Review: Mechanisms of change and between‐family differences in parenting interventions for children with ADHD – an individual participant data meta‐analysis
Source: J Child Psychol Psychiatry. 2025 Feb 5;66(9):1304–19. doi: 10.1111/jcpp.14120 (PMC12350818; doi:10.1111/jcpp.14120)
Supplement: Supplementary file 1 — Appendix S1. Changes from preregistration. Table S1. Baseline targeted moderated mediation analysis including the additional adolescent studies. Appendix S2. PRISMA checklist. Appendix S3. Search terms per database. Appendix S4. Supplementary tables. Table S4A. Outcome measures used per included study. Table S4B. Confirmatory factor model for masked and unmasked child behaviour. Table S4C. Correlation matrix between outcomes. Appendix S5. Supplementary figures. Figure S5A. PRISMA 2009 flow diagram. Figure S5B. Percentages of missing data on parenting outcomes within studies. Figure S5C. Percentages of missing data on child outcomes within studies. Figure S5D. Missing data patterns on the post‐intervention measures across studies. Figure S5E. Risk of bias studies. Figure S5F. Risk of bias per study. Figure S5G. Reverse mediation model. Appendix S6. Post hoc analysis results for the reverse mediation. Appendix S7. Sensitivity mediation analysis results on the multimodal interventions. [file JCPP-66-1304-s001.docx]

**Supporting Information**

**Appendix S1. Changes from Preregistration**Below we list differences from our pre-registered plans (AsPredicted # 126337), all of which were aimed at ensuring the most accurate and unbiased results:

1. Description of change: We excluded two eligible trials that were included in the original IPDMA dataset (*n* = 164, Sibley et al., 2014; Sibley et al., 2016) focusing exclusively on adolescents (average age >12).
2. Rationale: There might be differential mediation effects of parenting interventions for adolescents compared to younger children.
3. Effect of change on study results: None expected changes on the joint mediating effects of parenting behaviours and parent-child affection. However, there were no specific (moderated) mediating effects on child outcomes, in contrast to the main study results (see Table S3).
4. Description of change: We evaluated mediation utilising only the parent ratings of child behaviour because only three studies (*n* = 385) provided data on masked ADHD severity, and four studies (*n* = 200) on oppositional behaviour.
5. The model including the masked outcomes did not converge due to many missing patterns.
6. Results for treatment effects on masked outcomes would not be representative as the number of trials that provided data was limited and focused mainly on two interventions (i.e., Incredible Years and New Forest Parenting Programme).
7. Effect of change on study results: None expected changes on results, but it remains unknown if changes in parenting behaviour and parent-child affection may account for intervention effects on unmasked child outcomes.
8. Description of change: We planned to run a sensitivity analysis using masked observations for parenting practices and parent ratings of child outcomes to reduce the possibility of finding associations that might be confounded by informants’ attribution bias. However, there were insufficient trials with masked parenting measures to conduct these analyses.
9. Rationale: Masked observations of non-constructive parenting, constructive parenting, and parent-child affection were available in only 5 studies (*n* = 263), 7 studies (*n* = 506), and 6 studies (*n* = 458), respectively.
10. We are not aware if changes in parenting behaviour and parent-child affection may account for intervention effects on unmasked child outcomes. Also, as we depended on parental ratings of both their own and their child’s behaviour there might be inflated observed associations due to shared informant variance.
11. Effect of change on study results: We depended on parental ratings of both their own and their child’s behaviour, possibly causing inflated observed associations due to shared informant variance.
12. Description of change: We planned to rerun analyses separately for trials that examined a full parenting intervention versus trials that investigated a multi-modal intervention, including components in addition to the parenting component (e.g., child sessions, teacher sessions). We were able to conduct sensitivity analyses only in the subgroup of studies that tested a multimodal intervention, but not a full parenting intervention.
13. Rationale: Due to missing values (37 missing patterns), some pairwise combinations had less than 10% coverage.
14. Effect of change on study results: Study results are not affected, but we cannot compare mediation effects between the subgroups of studies. Also, because there were insufficient multimodal intervention trials (*k* = 7, *n* = 817) the results might not be reliable.

### ***Table S1 Baseline Targeted Moderated Mediation Analysis Including the Additional Adolescent Studies*** ***(n = 1885, k =21)***

|  |  | | Child Outcomes | | | | | |
| --- | --- | --- | --- | --- | --- | --- | --- | --- |
|  | |  | ADHD severity | | ODD behaviour | | Functional impairment | |
| Mediators | Indirect paths | | *β* (SE) | *p* | *β* (SE) | *p* | *β* (SE) | *p* |
| Non–constructive parenting | Specific indirect | | -0.022 (0.014) | 0.112 | -0.020 (0.012) | 0.106 | -0.024 (0.014) | 0.075 |
|  | Conditional indirect | | -0.016 (0.010) | 0.123 | -0.015 (0.009) | 0.100 | -0.018 (0.009) | 0.052 |
| Constructive parenting | Specific indirect | | 0.001(0.005) | 0.814 | 0.005 (0.003) | 0.096 | 0.004 (0.010) | 0.703 |
|  | Conditional indirect | | -0.002 (0.007) | 0.774 | -0.007 (0.004) | 0.108 | -0.005 (0.013) | 0.692 |
| Parent-child affection | Specific indirect | | -0.14 (0.009) | 0.137 | -0.019 (0.009) | 0.034 | -0.022 (0.012) | 0.068 |
|  | Conditional indirect | | 0.005 (0.006) | 0.389 | 0.007 (0.008) | 0.423 | 0.008 (0.009) | 0.361 |
| Total indirect | | | -0.035 (0.015) | 0.023 | -0.034 (0.013) | 0.010 | -0.043 (0.015) | 0.003 |
| Total | | | -0.192 (0.049) | <.001 | -0.108 (0.028) | <.001 | -0.142 (0.080) | 0.075 |

*Note.* Specific indirect effects represent the direct pathway from the intervention arm on each parenting variable (*a* paths) multiplied by the pathway of each parenting variable on each child outcome (*b* paths). The joint indirect effects represent the joint mediated pathways via all parenting variables in the mediation model. Total effects represent the direct *c* path from intervention to each child outcome in addition to the joint indirect effect. The conditional indirect effects of the moderated mediation model represent the parenting pathways that are moderated by the baseline levels of each parenting variable. *β =* Standardized path estimate; SE = Standard error. Intervention arm was modelled using a dummy code (parenting intervention: yes/no, with control condition as the reference group). The significance of effects was determined with a significance level of α = 0.05.

**Appendix S2. PRISMA Checklist**

***PRISMA-IPD Checklist of items to include when reporting a systematic review and meta-analysis of individual participant data (IPD)***

| **PRISMA-IPD**  **Section/topic** | **Item No** | **Checklist item** | **Reported on page** |
| --- | --- | --- | --- |
| **Title** | | | |
| Title | 1 | Identify the report as a systematic review and meta-analysis of individual participant data. | 1 |
| **Abstract** | | | |
| Structured summary | 2 | Provide a structured summary including as applicable: | 8-9 |
|  |  | **Background**: state research question and main objectives, with information on participants, Parenting interventions, comparators and outcomes. |  |
|  |  | **Methods**: report eligibility criteria; data sources including dates of last bibliographic search or elicitation, noting that IPD were sought; methods of assessing risk of bias. |  |
|  |  | **Results**: provide number and type of studies and participants identified and number (%) obtained; summary effect estimates for main outcomes (benefits and harms) with confidence intervals and measures of statistical heterogeneity. Describe the direction and size of summary effects in terms meaningful to those who would put findings into practice. |  |
|  |  | **Discussion:** state main strengths and limitations of the evidence, general interpretation of the results and any important implications. |  |
|  |  | **Other:** report primary funding source, registration number and registry name for the systematic review and IPD meta-analysis. |  |
| **Introduction** | | | |
| Rationale | 3 | Describe the rationale for the review in the context of what is already known. | 10 |
| Objectives | 4 | Provide an explicit statement of the questions being addressed with reference, as applicable, to participants, Parenting interventions, comparisons, outcomes and study design (PICOS). Include any hypotheses that relate to particular types of participant-level subgroups. | 13 |
| **Methods** | | | |
| Protocol and registration | 5 | Indicate if a protocol exists and where it can be accessed. If available, provide registration information including registration number and registry name. Provide publication details, if applicable. | 14 |
| Eligibility criteria | 6 | Specify inclusion and exclusion criteria including those relating to participants, Parenting interventions, comparisons, outcomes, study design and characteristics (e.g. years when conducted, required minimum follow-up). Note whether these were applied at the study or individual level i.e. whether eligible participants were included (and ineligible participants excluded) from a study that included a wider population than specified by the review inclusion criteria. The rationale for criteria should be stated. | 14-15 |
| Identifying studies - information sources | 7 | Describe all methods of identifying published and unpublished studies including, as applicable: which bibliographic databases were searched with dates of coverage; details of any hand searching including of conference proceedings; use of study registers and agency or company databases; contact with the original research team and experts in the field; open adverts and surveys. Give the date of last search or elicitation. | 14-15 |
| Identifying studies - search | 8 | Present the full electronic search strategy for at least one database, including any limits used, such that it could be repeated. | Appendix S3 |
| Study selection processes | 9 | State the process for determining which studies were eligible for inclusion. | 14-15 |
| Data collection processes | 10 | Describe how IPD were requested, collected and managed, including any processes for querying and confirming data with investigators. If IPD were not sought from any eligible study, the reason for this should be stated (for each such study). | 15 |
|  |  | If applicable, describe how any studies for which IPD were not available were dealt with. This should include whether, how and what aggregate data were sought or extracted from study reports and publications (such as extracting data independently in duplicate) and any processes for obtaining and confirming these data with investigators. |  |
| Data items | 11 | Describe how the information and variables to be collected were chosen. List and define all study level and participant level data that were sought, including baseline and follow-up information. If applicable, describe methods of standardising or translating variables within the IPD datasets to ensure common scales or measurements across studies. | 15-16 |
| IPD integrity | A1 | Describe what aspects of IPD were subject to data checking (such as sequence generation, data consistency and completeness, baseline imbalance) and how this was done. | 15 |
| Risk of bias assessment in individual studies. | 12 | Describe methods used to assess risk of bias in the individual studies and whether this was applied separately for each outcome. If applicable, describe how findings of IPD checking were used to inform the assessment. Report if and how risk of bias assessment was used in any data synthesis. | 17 |
| Specification of outcomes and effect measures | 13 | State all treatment comparisons of interests. State all outcomes addressed and define them in detail. State whether they were pre-specified for the review and, if applicable, whether they were primary/main or secondary/additional outcomes. Give the principal measures of effect (such as risk ratio, hazard ratio, difference in means) used for each outcome. | 15-16 & 17-20 |
| Synthesis methods | 14 | Describe the meta-analysis methods used to synthesise IPD. Specify any statistical methods and models used. Issues should include (but are not restricted to):   - Use of a one-stage or two-stage approach. - How effect estimates were generated separately within each study and combined across studies (where applicable). - Specification of one-stage models (where applicable) including how clustering of patients within studies was accounted for. - Use of fixed or random effects models and any other model assumptions, such as proportional hazards. - How (summary) survival curves were generated (where applicable). - Methods for quantifying statistical heterogeneity (such as I^2^ and τ^2^). - How studies providing IPD and not providing IPD were analysed together (where applicable). - How missing data within the IPD were dealt with (where applicable). | 17-20 |
| Exploration of variation in effects | A2 | If applicable, describe any methods used to explore variation in effects by study or participant level characteristics (such as estimation of interactions between effect and covariates). State all participant-level characteristics that were analysed as potential effect modifiers, and whether these were pre-specified. | 20 |
| Risk of bias across studies | 15 | Specify any assessment of risk of bias relating to the accumulated body of evidence, including any pertaining to not obtaining IPD for particular studies, outcomes or other variables. | 20 |
| Additional analyses | 16 | Describe methods of any additional analyses, including sensitivity analyses. State which of these were pre-specified. | 14, 17, Appendix S2 |
| **Results** | | | |
| Study selection and IPD obtained | 17 | Give numbers of studies screened, assessed for eligibility, and included in the systematic review with reasons for exclusions at each stage. Indicate the number of studies and participants for which IPD were sought and for which IPD were obtained. For those studies where IPD were not available, give the numbers of studies and participants for which aggregate data were available. Report reasons for non-availability of IPD. Include a flow diagram. | Figure S5A |
| Study characteristics | 18 | For each study, present information on key study and participant characteristics (such as description of Parenting interventions, numbers of participants, demographic data, unavailability of outcomes, funding source, and if applicable duration of follow-up). Provide (main) citations for each study. Where applicable, also report similar study characteristics for any studies not providing IPD. | Table 1 |
| IPD integrity | A3 | Report any important issues identified in checking IPD or state that there were none. | 15 |
| Risk of bias within studies | 19 | Present data on risk of bias assessments. If applicable, describe whether data checking led to the up-weighting or down-weighting of these assessments. Consider how any potential bias impacts on the robustness of meta-analysis conclusions. | 21 |
| Results of individual studies | 20 | For each comparison and for each main outcome (benefit or harm), for each individual study report the number of eligible participants for which data were obtained and show simple summary data for each Parenting intervention group (including, where applicable, the number of events), effect estimates and confidence intervals. These may be tabulated or included on a forest plot. | Table 1 for descriptives |
| Results of syntheses | 21 | Present summary effects for each meta-analysis undertaken, including confidence intervals and measures of statistical heterogeneity. State whether the analysis was pre-specified, and report the numbers of studies and participants and, where applicable, the number of events on which it is based. | 21-23 |
|  |  | When exploring variation in effects due to patient or study characteristics, present summary interaction estimates for each characteristic examined, including confidence intervals and measures of statistical heterogeneity. State whether the analysis was pre-specified. State whether any interaction is consistent across trials. |  |
|  |  | Provide a description of the direction and size of effect in terms meaningful to those who would put findings into practice. |  |
| Risk of bias across studies | 22 | Present results of any assessment of risk of bias relating to the accumulated body of evidence, including any pertaining to the availability and representativeness of available studies, outcomes or other variables. | 21 |
| Additional analyses | 23 | Give results of any additional analyses (e.g. sensitivity analyses). If applicable, this should also include any analyses that incorporate aggregate data for studies that do not have IPD. If applicable, summarise the main meta-analysis results following the inclusion or exclusion of studies for which IPD were not available. | Appendix S2, S6, S7, Figure S5D |
| **Discussion** | | | |
| Summary of evidence | 24 | Summarise the main findings, including the strength of evidence for each main outcome. | 23-24 |
| Strengths and limitations | 25 | Discuss any important strengths and limitations of the evidence including the benefits of access to IPD and any limitations arising from IPD that were not available. | 28-29 |
| Conclusions | 26 | Provide a general interpretation of the findings in the context of other evidence. | 24-28 |
| Implications | A4 | Consider relevance to key groups (such as policy makers, service providers and service users). Consider implications for future research. | 27-30 |
| **Funding** | | | |
| Funding | 27 | Describe sources of funding and other support (such as supply of IPD), and the role in the systematic review of those providing such support. | 31 |

**A1 – A3 denote new items that are additional to standard PRISMA items. A4 has been created as a result of re-arranging content of the standard PRISMA statement to suit the way that systematic review IPD meta-analyses are reported.**

© Reproduced with permission of the PRISMA IPD Group, which encourages sharing and reuse for non-commercial purposes

## **Appendix S3. Search Terms Per Database**

**PubMed**("Attention Deficit Disorder with Hyperactivity"[Mesh] OR ADHD[tiab] OR ADD[tiab] OR attention deficit*[tiab] OR hyperactiv*[tiab] OR hyperkinetic*[tiab] OR minimal brain deficit*[tiab] OR minimal brain dysfunction*[tiab])
**AND**

(("Psychotherapy"[Mesh] OR psychotherap*[tiab] OR psychological therap*[tiab] OR psychological intervent*[tiab] OR psychoeduca*[tiab] OR mentoring*[tiab] OR coaching*[tiab] OR mindful*[tiab] OR relax*[tiab] OR meditat*[tiab])

OR

((parent*[tiab] OR mother[tiab] OR father[tiab] OR teacher*[tiab] OR school*[tiab])

*AND*

(program*[tiab] OR train*[tiab] OR educa*[tiab] OR therapy*[tiab] OR therapies*[tiab] OR therapeu*[tiab]OR intervention*[tiab] OR coaching*[tiab] OR counseling*[tiab]))

OR

((behavio*[tiab] OR cognit*[tiab] OR “acceptance and commitment”[tiab] OR dialectica*[tiab])

*AND*

(program*[tiab] OR therapy*[tiab] OR therapies*[tiab] OR therapeu*[tiab] OR intervention*[tiab] OR treatment*[tiab] OR train*[tiab]))

OR

(psychosocial[tiab]

AND

(treatment*[tiab] OR therapy*[tiab] OR therapies*[tiab] OR therapeu*[tiab] OR train*[tiab] OR intervention*[tiab] OR program*[tiab]))

OR

((skill*[tiab] OR organization*[tiab] OR organisation*[tiab] OR planning*[tiab] OR play*[tiab])

AND

(train*[tiab] OR intervention*[tiab] OR program*[tiab])))

**AND**

("Adolescent"[Mesh] OR "Child"[Mesh] OR child*[tiab] OR school*[tiab] OR infan*[tiab] OR adolescen*[tiab] OR pediatri*[tiab] OR paediatr*[tiab] OR boy[tiab] OR boys[tiab] OR boyhood[tiab] OR girl[tiab] OR girls[tiab] OR girlhood[tiab] OR youth[tiab] OR youths[tiab] OR teen[tiab] OR teens[tiab] OR teenage*[tiab] OR puberty[tiab] OR preschool*[tiab] OR toddler*[tiab] OR juvenile*[tiab] OR kids[tiab])
**AND**

("Controlled Clinical Trial" [Publication Type] OR "Randomized Controlled Trials as Topic"[Mesh] OR "Follow-Up Studies"[Mesh] OR follow-up[tiab] OR followup[tiab] OR control group*[tiab] OR "Random Allocation"[Mesh] OR random*[tiab] OR trial[ti])

**EMBASE**

('attention deficit disorder'/exp OR (ADHD OR ADD OR ‘attention deficit*’

OR hyperactiv* OR hyperkinetic* OR ‘minimal brain deficit*’ OR ‘minimal brain dysfunction*’):ab,ti)

**AND**

('psychotherapy'/exp OR (psychotherap* OR ‘psychological therap*’ OR ‘psychological intervent*’ OR psychoeduca* OR mentoring* OR coaching* OR mindful* OR relax*):ab,ti)

OR

((((parent* OR mother OR father OR teacher* OR school*)

*AND*

(program* OR train* OR educa* OR therapy* OR therapies* OR therapeu*OR intervention* OR coaching* OR counseling*))

OR

((behavio* OR cognit* OR ‘acceptance and commitment’ OR dialectica*)

*AND*

(program* OR therapy* OR therapies* OR therapeu* OR intervention* OR treatment* OR train*))

OR

(psychosocial

AND

(treatment* OR therapy* OR therapies* OR therapeu* OR train* OR intervention* OR program*))

OR

((skill* OR organization* OR organisation* OR planning* OR play*)

AND

(train* OR intervention* OR program*))):ab,ti)

**AND**

('juvenile'/exp OR (child* OR school* OR infan* OR adolescen* OR pediatri* OR paediatr* OR boy OR boys OR boyhood OR girl OR girls OR girlhood OR youth OR youths OR teen OR teens OR teenage* OR puberty OR preschool* OR toddler* OR juvenile* OR kids):ab,ti)

**AND**

('controlled clinical trial'/exp OR 'randomization'/exp OR (‘control group*’ OR random*):ab,ti OR trial:ti)

**EBSCO PsycINFO**

(DE "Attention Deficit Disorder" OR DE "Attention Deficit Disorder with Hyperactivity" OR ADHD OR ADD OR “attention deficit*” OR hyperactiv* OR hyperkinetic* OR “minimal brain deficit*” OR “minimal brain dysfunction*”)

**AND**

(DE "Psychotherapy" OR DE "Child Psychotherapy" OR DE "Play Therapy" OR DE "Behavior Therapy" OR DE "Aversion Therapy" OR DE "Conversion Therapy" OR DE "Dialectical Behavior Therapy" OR DE "Exposure Therapy" OR DE "Implosive Therapy" OR DE "Reciprocal Inhibition Therapy" OR DE "Response Cost" OR DE "Systematic Desensitization Therapy" AND DE "Cognitive Behavior Therapy" OR DE "Cognitive Therapy" OR DE "Group Psychotherapy" OR DE "Neurotherapy" OR psychotherap* OR “psychological therap*” OR “psychological intervent*” OR psychoeduca* OR mentoring* OR coaching* OR mindful* OR relax*)

OR

((parent* OR mother OR father OR teacher* OR school*)

*AND*

(program* OR train* OR educa* OR therapy* OR therapies* OR therapeu*OR intervention* OR coaching* OR counseling*))

OR

((behavio* OR cognit* OR ‘acceptance and commitment’ OR dialectica*)

*AND*

(program* OR therapy* OR therapies* OR therapeu* OR intervention* OR treatment* OR train*))

OR

(psychosocial

AND

(treatment* OR therapy* OR therapies* OR therapeu* OR train* OR intervention* OR program*))

OR

((skill* OR organization* OR organisation* OR planning* OR play*)

AND

(train* OR intervention* OR program*))

**AND**

(AG (childhood OR adolescence) OR (child* OR school* OR infan* OR adolescen* OR pediatri* OR paediatr* OR boy OR boys OR boyhood OR girl OR girls OR girlhood OR youth OR youths OR teen OR teens OR teenage* OR puberty OR preschool* OR toddler* OR juvenile* OR kids))

**AND**

(“control group*” OR random* OR "controlled trial" OR "controlled study" OR "experimental study" OR "experimental design" OR TI trial)

**EBSCO CINAHL**

(MH "Attention Deficit Hyperactivity Disorder" OR "Attention Deficit Disorder with Hyperactivity" OR ADHD OR ADD OR “attention deficit*” OR hyperactiv* OR hyperkinetic* OR “minimal brain deficit*” OR “minimal brain dysfunction*”)

**AND**

(MH "Psychotherapy+" OR psychotherap* OR “psychological therap*” OR “psychological intervent*” OR psychoeduca* OR mentoring* OR coaching* OR mindful* OR relax*)

OR

((parent* OR mother OR father OR teacher* OR school*)

*AND*

(program* OR train* OR educa* OR therapy* OR therapies* OR therapeu*OR intervention* OR coaching* OR counseling*))

OR

((behavio* OR cognit* OR ‘acceptance and commitment’ OR dialectica*)

*AND*

(program* OR therapy* OR therapies* OR therapeu* OR intervention* OR treatment* OR train*))

OR

(psychosocial

AND

(treatment* OR therapy* OR therapies* OR therapeu* OR train* OR intervention* OR program*))

OR

((skill* OR organization* OR organisation* OR planning* OR play*)

AND

(train* OR intervention* OR program*))

**AND**

(AG child OR (child* OR school* OR infan* OR adolescen* OR pediatri* OR paediatr* OR boy OR boys OR boyhood OR girl OR girls OR girlhood OR youth OR youths OR teen OR teens OR teenage* OR puberty OR preschool* OR toddler* OR juvenile* OR kids))

**AND**

(MH "Clinical Trials+" OR “control group*” OR random* OR "controlled trial" OR "controlled study" OR "experimental study" OR "experimental design" OR TI trial)

**EBSCO ERIC**

(DE "Attention Deficit Disorder" OR DE "Attention Deficit Hyperactivity Disorder" OR ADHD OR ADD OR “attention deficit*” OR hyperactiv* OR hyperkinetic* OR “minimal brain deficit*” OR “minimal brain dysfunction*”)

**AND**

(DE "Psychotherapy" OR psychotherap* OR “psychological therap*” OR “psychological intervent*” OR psychoeduca* OR mentoring* OR coaching* OR mindful* OR relax*)

OR

((parent* OR mother OR father OR teacher* OR school*)

*AND*

(program* OR train* OR educa* OR therapy* OR therapies* OR therapeu*OR intervention* OR coaching* OR counseling*))

OR

((behavio* OR cognit* OR ‘acceptance and commitment’ OR dialectica*)

*AND*

(program* OR therapy* OR therapies* OR therapeu* OR intervention* OR treatment* OR train*))

OR

(psychosocial

AND

(treatment* OR therapy* OR therapies* OR therapeu* OR train* OR intervention* OR program*))

OR

((skill* OR organization* OR organisation* OR planning* OR play*)

AND

(train* OR intervention* OR program*))

**AND**

(child* OR school* OR infan* OR adolescen* OR pediatri* OR paediatr* OR boy OR boys OR boyhood OR girl OR girls OR girlhood OR youth OR youths OR teen OR teens OR teenager* OR puberty OR preschool* OR toddler* OR juvenile* OR kids))

**AND**

(“control group*” OR random* OR "controlled trial" OR "controlled study" OR "experimental study" OR "experimental design" OR TI trial)

**Web of Science**(TS=("Attention Deficit Disorder with Hyperactivity" OR ADHD OR ADD OR “attention deficit*” OR hyperactiv* OR hyperkinetic* OR “minimal brain deficit*” OR “minimal brain dysfunction*”))

**AND**

(TS=(psychotherap* OR psychotherap* OR “psychological therap*” OR “psychological intervent*” OR psychoeduca* OR mentoring* OR coaching* OR mindful* OR relax*

OR

((parent* OR mother OR father OR teacher* OR school*)

*AND*

(program* OR train* OR educa* OR therapy* OR therapies* OR therapeu*OR intervention* OR coaching* OR counseling*))

OR

((behavio* OR cognit* OR ‘acceptance and commitment’ OR dialectica*)

*AND*

(program* OR therapy* OR therapies* OR therapeu* OR intervention* OR treatment* OR train*))

OR

(psychosocial

AND

(treatment* OR therapy* OR therapies* OR therapeu* OR train* OR intervention* OR program*))

OR

((skill* OR organization* OR organisation* OR planning* OR play*)

AND

(train* OR intervention* OR program*))))

**AND**

(TS=(child* OR school* OR infan* OR adolescen* OR pediatri* OR paediatr* OR boy OR boys OR boyhood OR girl OR girls OR girlhood OR youth OR youths OR teen OR teens OR teenager* OR puberty OR preschool* OR toddler* OR juvenile* OR kids))

**AND**

(TS=(“clinical trial*” OR “control group*” OR random* OR "controlled trial" OR "controlled study" OR "experimental study" OR "experimental design"))

**Cochrane Central register of Controlled Trials** [*(CENTRAL)*](http://onlinelibrary.wiley.com/cochranelibrary/search?searchRow.searchOptions.searchProducts=clinicalTrialsDoi)

("Attention Deficit Disorder with Hyperactivity" OR ADHD OR ADD OR “attention deficit*” OR hyperactiv* OR hyperkinetic* OR “minimal brain deficit*” OR “minimal brain dysfunction*”)

**AND**

((psychotherap* OR “psychological therap*” OR “psychological intervent*” OR psychoeduca* OR mentoring* OR coaching* OR mindful* OR relax*)

OR

((parent* OR mother OR father OR teacher* OR school*)

*AND*

(program* OR train* OR educa* OR therapy* OR therapies* OR therapeu*OR intervention* OR coaching* OR counseling*))

OR

((behavio* OR cognit* OR ‘acceptance and commitment’ OR dialectica*)

*AND*

(program* OR therapy* OR therapies* OR therapeu* OR intervention* OR treatment* OR train*))

OR

((psychosocial)

AND

(treatment* OR therapy* OR therapies* OR therapeu* OR train* OR intervention* OR program*))

OR

((skill* OR organization* OR organisation* OR planning* OR play*)

AND

(train* OR intervention* OR program*)))

**AND**

(child* OR school* OR infan* OR adolescen* OR pediatri* OR paediatr* OR boy OR boys OR boyhood OR girl OR girls OR girlhood OR youth OR youths OR teen OR teens OR teenage* OR puberty OR preschool* OR toddler* OR juvenile* OR kids)

## **Appendix S4. Supplementary Tables**

### ***Table S4A Outcome Measures Used per Included Study***

|  | Unmasked ADHD | Masked ADHD | Unmasked oppositional behaviour | Masked oppositional behaviour | Unmasked functional impairment | Unmasked constructive parenting | Unmasked non-constructive parenting | Unmasked  Parent-child affection |
| --- | --- | --- | --- | --- | --- | --- | --- | --- |
| Aghebati, Gharraee, Hakim Shoshtari, and Gohari (2014) |  |  |  |  |  |  | PS negative parenting subscale | PBI care subscale |
| Daley and O'Brien (2013) | Dupaul rating scale | Direct observation of child overactivity and inattention time on task corrected for switches |  |  | SDQ parent rated impairment |  |  |  |
| Du Paul et al., (2018) |  |  |  |  | Colombia parent-rated impairment rating scale |  |  |  |
| Fabiano et al., (2012) | DBD ADHD rating scale |  | DBD ODD rating scale |  | IRS total parent-rated | APQ positive parenting subscale | APQ total of inconsistent discipline, poor monitoring, and corporal punishment subscales | APQ involvement subscale |
| Ferrin et al., (2014) | CPRS ADHD index |  | CPRS ODD index |  | CGI rated by masked clinician |  |  |  |
| Ferrin et al., (2020) | CPRS ADHD index |  | CPRS ODD index |  | CGI rated by masked clinician |  |  |  |
| Franke , Keown, and Sanders (2020) | CPRS ADHD index |  |  |  |  | PSDQ reasoning and induction subscale | PS negative parenting subscale | PSDQ total of warmth and good-natured/easy-going subscales |
| Herbert Harvey, Roberts, Wichowski, and Lugo‐Candelas (2013) | DBD ADHD rating scale |  | DBD ODD rating scale | Disruptive behaviour during a challenging parent-child interaction at home |  | CCNES total of expressive encouragement, emotion-focused reactions, and problem-focused reactions subscales | PS total of overreactivity, laxness, and verbosity subscales |  |
| Mautone et al., (2012) | SNAP ADHD rating scale |  | SNAP ODD rating scale | DPICS percentage of non-compliance to direct commands during cleanup task | IRS total parent-rated | Family Involvement Questionnaire total | PCRQ negative/ineffective discipline subscale | PCRQ total of warmth, disciplinary warmth, and good-natured/easy-going subscales |
| Mikami et al., (2010) | Conners CPRS ADHD index |  | CPRS ODD index |  |  |  |  |  |
| Power et al., (2012) | SNAP ADHD rating scale |  | SNAP ODD rating scale |  |  |  | PCRQ negative/ineffective discipline subscale | PCRQ total of warmth, disciplinary warmth, and good-natured/easy-going subscales |
| Pfiffner et al., (2007) | CSI ADHD severity |  | CSI ODD severity |  |  | APQ positive parenting subscale | APQ total of inconsistent discipline, poor monitoring, and corporal punishment subscales | APQ involvement subscale |
| Pfiffner et al., (2014) | CSI ADHD severity |  | CSI ODD severity |  | IRS total parent-rated | APQ positive parenting subscale | APQ total of inconsistent discipline, poor monitoring, and corporal punishment subscales | PCRQ total of warmth and personal relationship subscales |
| Pfiffner et al., (2016) | CSI ADHD severity |  | CSI ODD severity |  | IRS total parent-rated | APQ positive parenting subscale | APQ total of inconsistent discipline, poor monitoring, and corporal punishment subscales | APQ involvement subscale |
| Shimabukuro et al., (2020) | SNAP ADHD rating scale |  | SNAP ODD rating scale |  |  |  | PS total of overreactivity, laxness, and verbosity subscales |  |
| Sonuga-Barke et al., (2018) | SNAP ADHD rating scale | Direct observation of child over-activity and inattention time on task corrected for switches | SNAP ODD rating scale |  |  |  |  |  |
| Thompson et al., (2009) | Werry Weis Peters total score | Direct observation of child over-activity and inattention time off task |  | GIPCI-R total disruptive and non-compliance during free play, challenging tasks, and cleanup tasks |  | PPI total of praise, appropriate discipline, positive verbal discipline, monitoring, and clear expectations subscales | PPI total of harsh/ inconsistent discipline and physical punishment subscales |  |
| Van Den Hoofdakker et al., (2007) | CPRS ADHD index |  | CPRS ODD index |  |  |  |  | PSI parent domain attachment subscale |
| Webster Stratton et al., (2011) | CPRS ADHD index |  | CPRS ODD index | DPICS total disruptive behaviour during free play and challenging task |  | PPI total of praise, appropriate discipline, positive verbal discipline, monitoring, and clear expectations subscales | PPI total of harsh/ inconsistent discipline and physical punishment subscales |  |
| *Note.* APQ = Alabama Parenting Questionnaire, CCNES = Coping With Children's Negative Emotion Scale, CGI = Clinical Global Impression Scale, CPRS = Conners Parent Rating Scale, CSI = Child Symptom Inventory, DBD = Disruptive Behaviour Disorder, DPICS = Dyadic Parent-Child Interaction Coding System, GIPCI-R = Global Impressions Of Parent–Child Interactions-Revised, ODD = Oppositional Defiant Disorder, PCRQ = Parent-Child Relationship Questionnaire, PPI = Parenting Practices Inventory, PS = Parenting Scale, PSDQ = Parental Style Dimension Questionnaire, PSI = Parent Stress Index, SDQ = Strengths And Difficulties Questionnaire, SNAP = Swanson, Nolan And Pelham Questionnaire, SSIS = Social Skills Improvement System, IRS = Impairment Rating Scale | | | | | | | | |

### ***Table S4B Confirmatory Factor Model for Masked and Unmasked Child Behaviour***

| Model fit | Chi-square (*df*) | *p* | RMSEA (90% CI) | CFI | TLI |
| --- | --- | --- | --- | --- | --- |
|  | 78.663 (7) | < 0.01 | 0.075 (0.061, 0.090) | 0.834 | 0.858 |
| Factors | Variance (SE) | *p* | Covariance (SE) | *p* | |
| ADHD severity | 0.084 (0.054) | 0.118 | 0.393 (0.024) | <0.00 | |
| Oppositional behaviour | 0.075 (0.077) | 0.331 |  |  |  |

*Note.* CFI = Comparative fit index; *df =* Degrees of freedom; RMSEA = Root-mean-square errors of approximation; SE = Standard error; TLI = Tucker–Lewis index.

### ***Table S4C Correlation Matrix Between Outcomes***

| Variable |  | 1. | 2. | 3. | 4. | 5. | 6. |
| --- | --- | --- | --- | --- | --- | --- | --- |
| 1. Child ADHD severity | *r* | 1 | 0.571^***^ | 0.259^***^ | 0.193^***^ | -0.053 | -0.124^***^ |
|  | N | 1474 | 1356 | 645 | 867 | 654 | 781 |
| 1. Child oppositional behaviour | *r* |  | 1 | 0.202^***^ | 0.264^***^ | -0.096^**^ | -0.262^***^ |
|  | N |  |  | 605 | 794 | 583 | 736 |
| 1. Functional impairment | *r* |  |  | 1 | 0.135^**^ | -0.058 | -0.108^**^ |
|  | N |  |  | 646 | 450 | 465 | 465 |
| 1. Non-constructive parenting | *r* |  |  |  | 1 | -0.152^***^ | -0.213^***^ |
|  | N |  |  |  | 899 | 643 | 700 |
| 1. Constructive parenting | *r* |  |  |  |  | 1 | 0.573^***^ |
|  | N |  |  |  |  | 658 | 508 |
| 1. Parent-child affection | *r* |  |  |  |  |  | 1 |
|  | N |  |  |  |  | 508 | 811 |

Note. All variables are measured at post-intervention and are transformed into z-scores with the baseline standard deviation. *r* = Pearson’s Correlation; N = Sample Size. ^*^ p < .05, ^**^ p < .01, ^***^ p < .001.

## **Appendix S5. Supplementary Figures**

### ***Figure S5A PRISMA 2009 Flow Diagram*** *Note.* A comparison between studies providing data versus studies not providing data for the original IPDMA revealed no significant differences in intervention effect sizes on ADHD severity and functional impairment, but significantly lower effects on oppositional behaviour. Studies not providing data had a slightly larger percentage of male participants, smaller sample sizes and an earlier publication date (see Groenman et al., 2022).

## Included

Full-text articles excluded:

Other publication type (*k =* 72)

No RCT/ wrong design (*k =* 55)

Other intervention (*k =* 55)

Re-analysis (*k =* 38)

Other population (*k =* 29)

Foreign language (*k =* 14)

Medication (*k =* 20)

Other control group (*k =* 10)

Other outcome (*k =* 16)

Studies included in quantitative synthesis (IPDMA)
(*k =* 19)

## Eligibility

## Identification

## Screening

Additional records identified through other sources
(*k =* 2)

Records after duplicates removed
(*k =* 13053)

Title and abstract screened
(*k =* 13053)

Records excluded
(*k =* 12723)

Full-text articles assessed for eligibility
(*k =* 330)

Records identified through database searching
(*k =* 23023)

### ***Figure S5B Percentages of Missing Data on Parenting Outcomes within Studies***

| **Study_ID** | **N** | **Constructive parenting** | | **Non-constructive parenting** | | **Parent-child affection** | |
| --- | --- | --- | --- | --- | --- | --- | --- |
|  |  | **Missing baseline** | **Attrition** | **Missing baseline** | **Attrition** | **Missing baseline** | **Attrition** |
| Aghebati et al., 2014 | 27 |  |  | 0 | 0 | 0 | 0 |
| Daley and O'Brien (2013) | 43 |  |  |  |  |  |  |
| DuPaul et al., 2018 | 45 |  |  |  |  |  |  |
| Fabiano et al., 2012 | 55 | 0 | 11 | 0 | 11 | 0 | 11 |
| Ferrin et al., 2020 | 69 |  |  |  |  |  |  |
| Ferrin et al., 2014 | 81 |  |  |  |  |  |  |
| Franke et al., 2020 | 53 | 0 | 15 | 0 | 13 | 0 | 13 |
| Herbert et al., 2013 | 31 | 3 | 3 | 3 | 0 |  |  |
| Mautone et al., 2012 | 61 | 13 | 16 | 13 | 20 | 13 | 18 |
| Mikami et al., 2010 | 62 |  |  |  |  |  |  |
| Pfiffner et al., 2007 | 69 | 17 | 1 | 19 | 1 | 17 | 1 |
| Pfiffner et al., 2014 | 199 | 1 | 4 | 4 | 3 | 1 | 3 |
| Pfiffner et al., 2016 | 135 | 1 | 6 | 7 | 4 | 1 | 5 |
| Power et al., 2012 | 199 |  |  | 6 | 5 | 6 | 4 |
| Shimabukuro et al., 2020 | 52 |  |  | 2 | 4 |  |  |
| Sonuga-Barke et al., 2018 | 306 |  |  |  |  |  |  |
| Thompson et al., 2009 | 41 | 2 | 29 | 2 | 29 |  |  |
| Van den Hoofdakker et al., 2007 | 94 |  |  |  |  | 0 | 0 |
| Webster Stratton et al., 2011 | 99 | 2 | 6 | 2 | 6 |  |  |

*Note.* Red cells indicate data missing completely within studies and blue cells indicate no missing data. Green cells indicate <10% missing data, yellow cells indicate >10% missing data, and orange cells >20% missing data , and on the baseline measures, and on post-intervention related to attrition.

### ***Figure S5C Percentages of Missing Data on Child Outcomes within Studies***

|  |  | **ADHD severity** | | **Oppositional behaviour** | | **Impairment** | |
| --- | --- | --- | --- | --- | --- | --- | --- |
| **Study_ID** | **N** | **Missing baseline** | **Attrition** | **Missing baseline** | **Attrition** | **Missing baseline** | **Attrition** |
| Aghebati et al., 2014 | 27 |  |  |  |  |  |  |
| Daley and O'Brien (2013) | 43 |  |  |  |  | 7 | 0 |
| DuPaul et al., 2018 | 45 | 0 | 13 | 0 | 13 | 0 |  |
| Fabiano et al., 2012 | 55 |  |  | 0 | 11 | 2 | 9 |
| Ferrin et al., 2020 | 69 | 0 | 0 | 0 | 0 | 1 | 0 |
| Ferrin et al., 2014 | 81 | 2 | 0 | 2 | 0 | 20 | 0 |
| Franke et al., 2020 | 53 | 0 | 17 |  |  |  |  |
| Herbert et al., 2013 | 31 | 3 | 0 | 3 |  |  |  |
| Mautone et al., 2012 | 61 | 13 | 16 | 13 | 18 | 13 | 16 |
| Mikami et al., 2010 | 62 | 2 |  | 2 |  |  |  |
| Pfiffner et al., 2007 | 69 | 0 | 3 | 0 | 3 |  |  |
| Pfiffner et al., 2014 | 199 | 0 | 3 | 1 | 2 | 2 | 1 |
| Pfiffner et al., 2016 | 135 | 0 | 3 | 0 | 3 | 0 | 3 |
| Power et al., 2012 | 199 | 5 | 4 | 7 | 3 |  |  |
| Shimabukuro et al., 2020 | 52 | 0 | 8 | 0 | 8 |  |  |
| Sonuga-Barke et al., 2018 | 306 | 0 | 20 | 0 | 20 |  |  |
| Thompson et al., 2009 | 41 | 0 | 29 |  |  |  |  |
| Van den Hoofdakker et al., 2007 | 94 | 0 | 0 | 0 | 0 |  |  |
| Webster Stratton et al., 2011 | 99 | 2 | 7 | 2 | 7 |  |  |

*Note.* Red cells indicate data missing completely within studies and blue cells indicate no missing data. Green cells indicate <10% missing data, yellow cells indicate >10% missing data, and orange cells >20% missing data , and on the baseline measures, and on post-intervention related to attrition.

### ***Figure S5D Missing Data Patterns on the Post-Intervention Measures Across Studies***

*
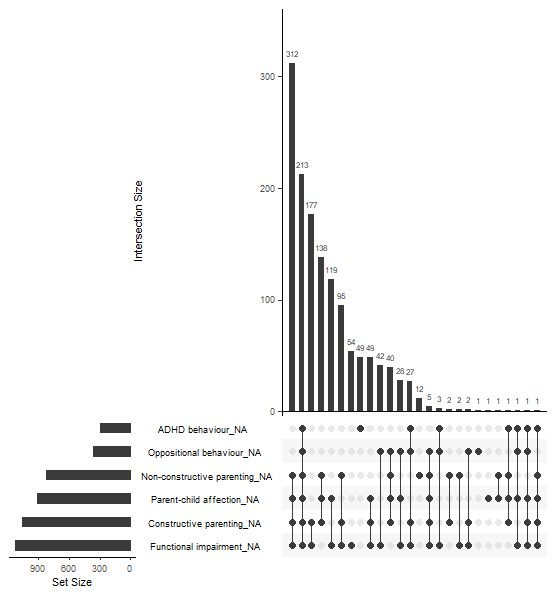
*

*Note.* The bar chart on the left side of the figure indicates the number of individuals that have missing values in each variable. The matrix of dots, next to this, illustrates the combinations of missing data patterns across the variables in our dataset. The dots that are connected by lines indicate which variables are missing together, and the intersection size including the histogram shows the number of individuals that have missing data for that combination of variables.

### ***Figure S5E Risk of Bias Studies***

**
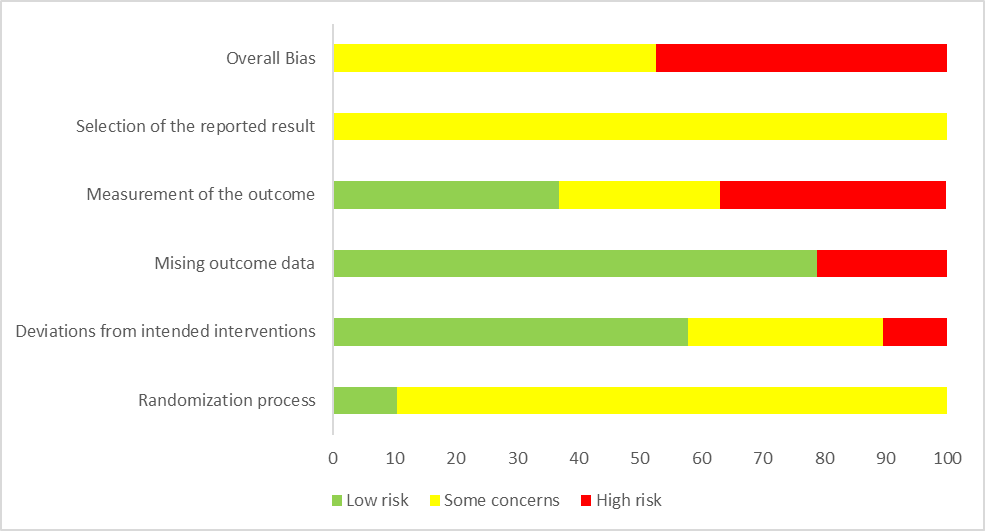
**

*Note.* Separate panels for those studies providing data and those not are available in Groenman et al., (2022). A comparison showed that in the studies not providing data, there were slightly more problems in the randomisation process. Overall, 28% of studies had a high risk of bias on 2 or more items. The percentage of risk-of-bias violations was not associated with any of the results on the outcome

measures (ADHD p = .23, oppositional behaviour p = .92, functional impairment p = .75).

### ***Figure S5F Risk of Bias Per Study***

*Note.* Our ratings on domain 4.5. distinguished the risk of bias in trials that did not include a masked measure but used an active control (assessed as ‘Some concerns’) versus a passive control comparison (assessed as ‘High’). This decision was made because not masking the outcome assessors is inherent to psychosocial research as it is impossible to hide from participants that they have received an intervention. The closest equivalent to a placebo comparison, which allows for the most rigorous control of bias in medical trials, is active group comparisons. Therefore, for these trials, knowledge of the intervention condition could confound the true effect of treatment, but there is less reason to believe that it did more in one condition over the other.

### ***Figure S5G Reverse Mediation Model***

*Pathways of Change in Parenting Outcomes Through Change in Child Behaviour and Functional Impairment*


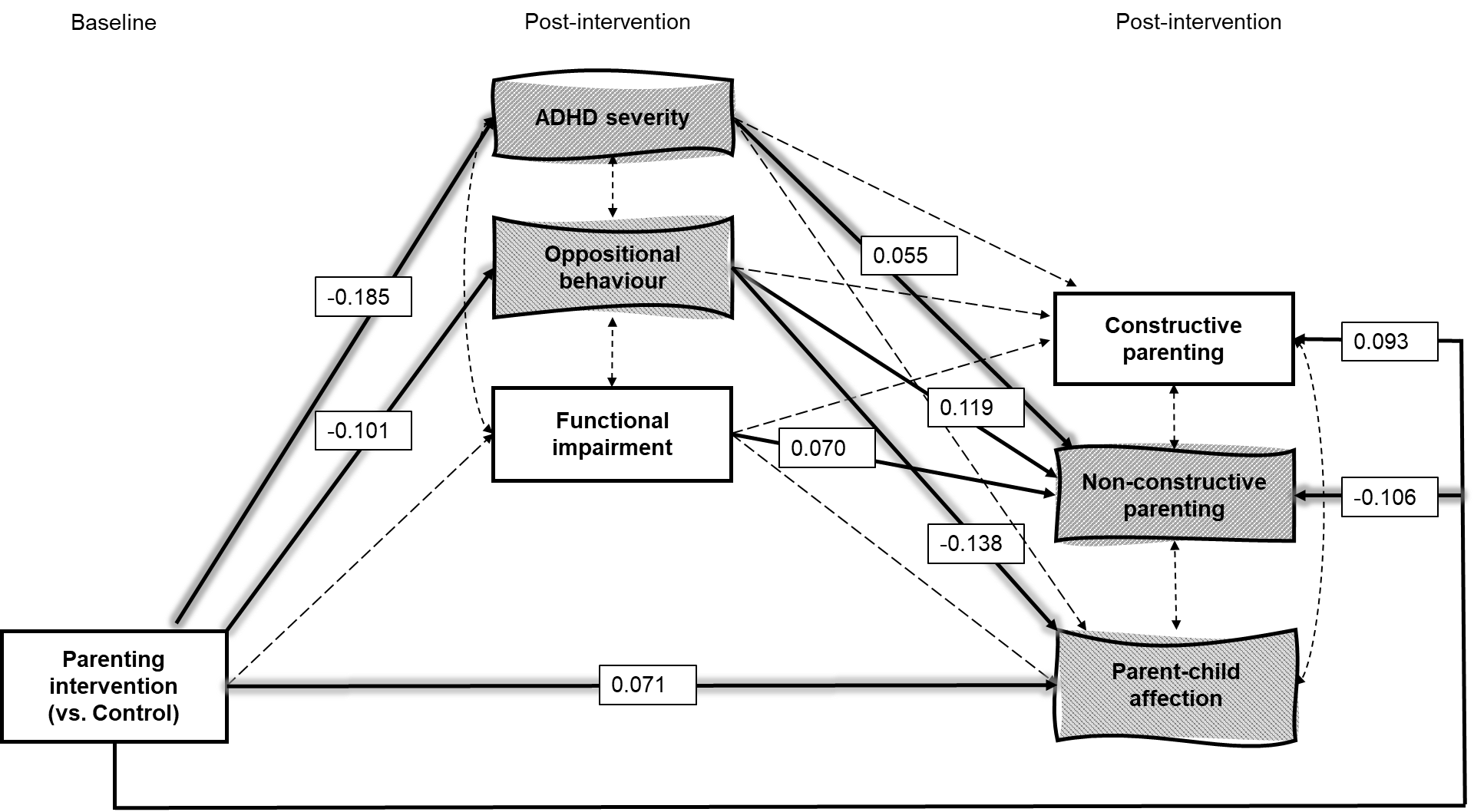


*Note.* Dashed double-headed arrows represent covariances between error terms. Dashed arrows depict non-significant paths, while solid arrows depict significant paths, with their standardised estimates in squares. Corresponding statistics for all paths are provided in Table 2. The grey wavy squares with glowing arrows highlight the specific mediated pathways for changes in non-constructive parenting through changes in ADHD severity and for parent-child affection through changes in oppositional behaviour. All post-intervention measures were controlled for their baseline values to reflect change; these controls are not depicted in the figure for the sake of readability.

**Appendix S6. Post-hoc Analysis Results for the Reverse Mediation**

|  |  | Parenting Outcomes | | | | | |
| --- | --- | --- | --- | --- | --- | --- | --- |
|  |  | Constructive parenting | | Non–constructive parenting | | Parent-child affection | |
| Mediators | Indirect paths | *β* (SE) | *p* | *β* (SE) | *p* | *β* (SE) | *p* |
| ADHD severity | Specific indirect | 0.005 (0.005) | 0.353 | -0.010 (0.004) | 0.012 | 0.008 (0.006) | 0.232 |
| Oppositional behaviour | Specific indirect | 0.003 (0.003) | 0.386 | -0.012 (0.005) | 0.011 | 0.014 (0.006) | 0.014 |
| Functional impairment | Specific indirect | 0.003 (0.005) | 0.519 | -0.010 (0.007) | 0.137 | 0.010 (0.009) | 0.265 |
| Total indirect | | 0.010 (0.005) | 0.045 | -0.032 (0.010) | 0.002 | 0.031 (0.016) | 0.031 |
| Total | | 0.103 (0.005) | 0.519 | -0.138 (0.058) | 0.018 | 0.102 (0.032) | 0.002 |

*Note.* Specific indirect effects represent the direct effect from the intervention arm to each parenting mediator (*a* paths) multiplied by the effect of each mediator on each child outcome (*b* paths). Conditional indirect effects represent the specific mediated effects moderated by the baseline levels of the corresponding parenting mediator. The total indirect effects represent the joint mediating effect of all mediators. Total effects represent the direct *c* path from intervention to each child outcome in addition to the total indirect effect. *β =* Standardized path estimate; SE = Standard error. Intervention arm was modelled using a dummy code (parenting intervention: yes/no, with control condition as the reference group). The significance of effects was determined with a significance level of α = 0.05.

**Appendix S7. Sensitivity Mediation Analysis Results on the Multimodal Intervention Studies (*n* = 762, *k* =6),**

|  |  | Child Outcomes | | | | | |
| --- | --- | --- | --- | --- | --- | --- | --- |
|  |  | ADHD severity | | Oppositional behaviour | | Functional impairment | |
| Mediators | Indirect paths | *β* (SE) | *p* | *β* (SE) | *p* | *β* (SE) | *p* |
| Constructive parenting | Specific indirect | -0.003 (0.005) | 0.466 | -0.000 (0.003) | 0.958 | -0.011 (0.008) | 0.167 |
| Non–constructive parenting | Specific indirect | -0.016 (0.013) | 0.219 | -0.014 (0.012) | 0.256 | -0.017 (0.012) | 0.155 |
| Parent-child affection | Specific indirect | -0.002 (0.002) | 0.322 | -0.007 (0.005) | 0.145 | -0.008 (0.004) | 0.035 |
| Total indirect | | -0.021 (0.014) | 0.130 | -0.021 (0.012) | 0.084 | -0.036 (0.015) | 0.017 |
| Total | | -0.240 (0.076) | 0.002 | -0.116 (0.040) | 0.004 | -0.231 (0.040) | 0.000 |

*Note.* Specific indirect effects represent the direct effect from the intervention arm to each parenting mediator (*a* paths) multiplied by the effect of each mediator to each child outcome (*b* paths). The total indirect effects represent the joint mediating effect of all mediators. Total effects represent the direct *c* path from intervention to each child outcome in addition to the total indirect effect. *β =* Standardized path estimate; SE = Standard error. Intervention arm was modelled using a dummy code (parenting intervention: yes/no, with control condition as the reference group). The significance of effects was determined with a significance level of α = 0.05.
